# Specific functions of TET1 and TET2 in regulating mesenchymal cell lineage determination

**DOI:** 10.1186/s13072-018-0247-4

**Published:** 2019-01-03

**Authors:** Dimitrios Cakouros, Sarah Hemming, Kahlia Gronthos, Renjing Liu, Andrew Zannettino, Songtao Shi, Stan Gronthos

**Affiliations:** 1 Mesenchymal Stem Cell Laboratory, Adelaide Medical School, Faculty of Health and Medical Sciences, University of Adelaide, Adelaide, SA 5000 Australia; 2 South Australian Health and Medical Research Institute, Adelaide, SA 5000 Australia; 3 Agnes Ginges Laboratory for Diseases of the Aorta, Centenary Institute for Cancer Medicine and Cell Biology, University of Sydney, Sydney, NSW 2042 Australia; 4 Multiple Myeloma Laboratory, Adelaide Medical School, Faculty of Health and Medical Sciences, University of Adelaide, Adelaide, SA 5000 Australia; 5 Department of Anatomy and Cell Biology, School of Dental Medicine, University of Pennsylvania, Philadelphia, PA 19104 USA

**Keywords:** Mesenchymal stem cells, Osteogenesis, Epigenetics, Hydroxymethylation, TET, Osteoporosis

## Abstract

**Background:**

The 5 hydroxymethylation (5hmC) mark and TET DNA dioxygenases play a pivotal role in embryonic stem cell differentiation and animal development. However, very little is known about TET enzymes in lineage determination of human bone marrow-derived mesenchymal stem/stromal cells (BMSC). We examined the function of all three TET DNA dioxygenases, responsible for DNA hydroxymethylation, in human BMSC cell osteogenic and adipogenic differentiation.

**Results:**

We used siRNA knockdown and retroviral mediated enforced expression of TET molecules and discovered TET1 to be a repressor of both osteogenesis and adipogenesis. TET1 was found to recruit the co-repressor proteins, SIN3A and the histone lysine methyltransferase, EZH2 to osteogenic genes. Conversely, TET2 was found to be a promoter of both osteogenesis and adipogenesis. The data showed that TET2 was directly responsible for 5hmC levels on osteogenic and adipogenic lineage-associated genes, whereas TET1 also played a role in this process. Interestingly, TET3 showed no functional effect in BMSC osteo-/adipogenic differentiation. Finally, in a mouse model of ovariectomy-induced osteoporosis, the numbers of clonogenic BMSC were dramatically diminished corresponding to lower trabecular bone volume and reduced levels of TET1, TET2 and 5hmC.

**Conclusion:**

The present study has discovered an epigenetic mechanism mediated through changes in DNA hydroxymethylation status regulating the activation of key genes involved in the lineage determination of skeletal stem cells, which may have implications in BMSC function during normal bone regulation. Targeting TET molecules or their downstream targets may offer new therapeutic strategies to help prevent bone loss and repair following trauma or disease.

**Electronic supplementary material:**

The online version of this article (10.1186/s13072-018-0247-4) contains supplementary material, which is available to authorized users.

## Introduction

DNA methylation and hydroxymethylation have been shown to regulate stem cell maintenance and differentiation via the activation or suppression of pluripotency and lineage-specific gene expression [1]. Although DNA methylation was one of the first epigenetic modifications studied, 5 hydroxymethylation has only recently been identified as an intermediate step in the process of DNA demethylation. Rao and colleagues [2] were the first to describe that ten-eleven translocation-1 (TET1) proteins can catalyse conversion of 5 methylcytosine (5mC) into 5 hydroxymethylcytosine (5hmC), as an intermediate step to the removal of the methylation mark on DNA, where 5hmC opposes the repressive effects of 5mC and can prevent methylation of DNA [2]. The TET protein family are comprised of 2-oxoglutarate and iron-dependent dioxygenases, where TET1 is mainly expressed by ESC, while TET2 and TET3 are more ubiquitously expressed across different cell lineages [2, 3]. ESC studies have shown that a key event in differentiation is the repression of pluripotency factors such as Oct4 and Nanog partly by DNA methylation [4]. *TET1*-deficient ESC display impaired self-renewal, diminished proliferation, with reduced *Nanog* expression coinciding with increased DNA methylation of its promoter [5]. Hematopoietic stem cells (HSC) derived from conditional *TET2* knockout mice display an enhanced ability to reconstitute haematopoiesis in vivo but prevent HSC from undergoing differentiation, providing evidence that TET2 restricts aberrant self-renewal and expansion of hematopoietic stem cells (HSC) [6]. In support of this notion, one of the most common mutations in myeloid malignancies is present in *TET2*, where *TET2*-deficient HSC show a skewed differentiation towards myeloid lineages at the expense of lymphoid lineages [7, 8]. Recent studies of global *TET1* and conditional *TET2* knockout mice in the mesenchyme lineage showed impaired bone-forming capacity in BMSC [9]. In other systems, conditional knockout of *TET2* in smooth muscle demonstrated that TET2 is essential for smooth muscle cell differentiation and that loss of *TET2* expression results in de-differentiation [10]. Other studies reported that TET1 and TET2 mediate Foxp3 demethylation to drive regulatory T cell differentiation [11]. A combined loss of *TET1* and *TET2* results in depleted 5hmC levels [12], with most mice exhibiting midgestation defects and perinatal lethality. Triple knockouts of *TET1/TET2/TET3* display a complete loss of 5hmC and increase in 5mC [13]. Differentiation of embryoid bodies is grossly impaired with a lack of mesoderm and endoderm markers. Global knockdown of all three *TET* molecules identified 1072 downregulated genes and 729 upregulated genes, illustrating that TET proteins can activate or repress transcription [13]. Furthermore, reprogramming of fibroblasts into iPSC results in increased levels of *TET1* and *TET2* and a decrease in *TET3* [14].

Bone marrow mesenchymal stem/stromal cells (BMSC) exhibit the capacity for multi-lineage differentiation and self-renewal [15–19]. BMSC maintenance and cell fate determination have previously been shown to be mediated, in part, by the activity of the histone 3 lysine 4 (H3K4) methyltransferase, MLL1/2 [20], the H3K27 methyltransferase, Ezh2 [21], and associated demethylases, KDM6A [21] and KDM6B [22–24], via the regulation of key lineage-associated transcription factors [25–27]. In an effort to further identify epigenetic enzymes involved in BMSC lineage determination and growth, we examined the function of TET DNA hydroxymethylases in human BMSC lineage determination. Previous studies have shown that TET1 can influence recruitment of Ezh2 to promoters [28], and plays a role in stem cell self renewal. In this study, we have identified a function role for both *TET1* and *TET2* in regulating human BMSC differentiation, by acting on genes involved in lineage determination. Moreover, we discovered that the expression of *TET1* and *TET2* is grossly deregulated in osteoporosis leading to deregulated 5hmC levels on promoters of genes controlling stem cell renewal and lineage determination in osteoporosis.

## Materials and methods

### Cell culture and antibodies

Human BMSC were derived from bone marrow aspirates from posterior iliac crest of normal adult volunteers after obtaining informed consent according to procedures approved by the Human Ethics Committee of the Royal Adelaide Hospital, South Australia (protocol# 940911a). Immunoselected STRO-1^+^ BMSC were cultured in regular growth medium as previously described [29].

### In vitro differentiation assays

Human BMSC were cultured in either normal growth conditions, osteogenic inductive conditions (control growth media + 10^−7^M dexamethasone, 10 mM HEPES buffer and 2.6 mM potassium phosphate) or adipogenic inductive conditions (control growth media + 0.5 mM, methylisobutylmethylxanthine, 0.5 μM hydrocortisone and 60 μM indomethacin) for up to 28 days as previously described [18]. Mineralised bone matrix formation was identified with Alizarin red (Sigma Aldrich Inc.) staining [29]. Extracellular calcium was measured in triplicate samples and normalised to DNA content per well as previously described [29]. Lipid formation was assessed and quantitation of lipid was performed by Nile red (Sigma Aldrich Inc, St Louis, MO) fluorescence staining, normalised to DAPI (Invitrogen/Life Technologies Australia, Mulgrave, VIC, AUS) stained nuclei per field of view in triplicate wells as previously described [29, 30].

### Lentiviral transduction

Lentiviral transductions were performed by transfecting 5 μg of Lv105 (cat:Ex-Neg-Lv105; Geneocoepia, Rockville, MD), Lv105-TET2 [10], Lv231 (Ex-Neg-Lv231), Lv231-TET1 (Ex-E2856-Lv231) into HEK293 T cells together with 5 μg of packaging vector psPAX2 and VsVG using lipofectamine 2000 (Life Technologies, Carlsbad, CA). After 48 h, 5 × 10^4^ BMSC were infected with the supernatant for the HEK293 T cells three times every 12 h in the presence of 4 mg/ml polybrene. Transduced BMSC were selected with 1 μg/ml puromycin for 7 days and then maintained in 200 ng/ml puromycin.

### siRNA knockdown

BMSC were transfected with 12 pmol siRNA targeting either TET1 (s37193; Ambion, Foster City, California), TET2 (cat: s29443), TET3 (s47238) or scramble control siRNA (AM4613), with RNAiMax lipofectamine (56532) in 100 μl media without foetal calf serum for 20 min. After 72 h, the media were replaced with either control growth media, osteogenic or adipogenic inductive media [29, 31, 32].

### RNA extractions, cDNA synthesis and real-time PCR

Total RNA from approximately 1.5 × 10^5^ human BMSC was isolated using Trizol (Invitrogen, Grand Island, NY) according to manufacturers’ instructions. cDNA and real-time PCR was performed as previously described in triplicate [32]. Changes in gene expression were calculated relative to β-actin using the 2^−dCT^ method.

### Oligonucleotides and primers

#### Real-time primers

*β*-*ACTIN*, F: 5′gatcattgctcctcctgagc3′, R: 5′gtcatagtccgcctagaagcat3′. *PPARγ2*, F: 5′ctcctattgacccagaaagc3′, R: 5′tcaaaggagtgggagtggtc3′; *ADIPSIN*, F: 5′gacaccatcgaccacgac3′, R: 5′ccacgtcgcagagagttc3′. *C/EBPα,* F: 5′gggcaaggccaagaagtc3′; R: 5′ttgtcactggtcagctccag3′. *LEPTIN,* F: 5′gaaccctgtgcggattcttgt3′; R: 5′tccatcttggataaggtcaggat3′. *RUNX2*, F: 5′gtggacgaggcaagagtttca-3′, R: 5′catcaagcttctgtctgtgcc3′. *OSTEOPONTIN*, F: 5′acatccagtaccctgatgctacag3′, R: 5′gtgggtttcagcactctggt3′. *OSTEOCALCIN*, F: 5′atgagagccctcacactcctcg3′, R: 5′gtcagccaactcgtcacagtcc3′. *TET1*, F: 5′gcagcgtacaggccaccact3′; R: 5′agccggtcggccattggaag3′. *TET2*, F: 5′ttcgcagaagcagcagtgaagag3′; R: 5′agccagagacagcgggattcctt3′. *TET3,* F: 5′gacgagaacatcggcggcgt3′; R: 5′gtggcagcggttgggcttct3′.

#### ChIP primers

*RUNX2* TSS F: 5′aggccttaccacaagccttt3′, R: 5′agaaagtttgcaccgcactt3′; *RUNX2* Exon F: 5′gcaaaatgagcgacgtgag3′, R: 5′acaggaagttggggctgtc3′; *RUNX2* Intron F: 5′cgattcaagagctgctcaca3′, R: 5′cctgttttgccgtcaatttt3′; *RUNX2* UTR F: 5′tttgcactgggtcatgtgtt3′, R: 5′tggctgcattgaaaagactg3′; *BMP2* TSS F: 5′atgcggagcacctactgc3′, R: 5′ccgcatcactctgccttact3′; *BMP2* Exon F: 5′cttctagcgttgctgcttcc3′, R: 5′agtgcctgcgatacaggtct3′; *BMP2* Intron F: 5′aaacaggccaaacacagtcc3′, R: 5′agccagggtctcagaacaga3′; *BMP2* UTR F: 5′tgcaggaaagtgaatgatgg3′, R: 5′tgcataattttgctgcgtgt3′; *PPARγ* TSS F: 5′agcaaacgacaccaggtagc3′, R: 5′ggcacccgtactctgacct3′; *PPARγ* Exon F: 5′gctgtgcaggagatcacaga3′, R: 5′gggctccataaagtcaccaa3′; *PPARγ* Intron F: 5′tcctctccagcgtctgtttt3′, R: 5′cccatctgacaaagggctaa3′; *PPARγ* UTR F: 5′cacagatccaccgtttcctt3′, R: 5′acacggtgaaaccctgtctc3′; *ADIPSIN* TSS F: 5′cctccaccctcataaaagca3′, R: 5′gcgttcagagccttccatta3′; *ADIPSIN* Exon F: 5′ctggggcatagtcaaccac3′, R: 5′atcaagcgctcggtgatg3′; *ADIPSIN* Intron F: 5′ggaagagaaggggtcctgag3′, R: 5′tccaagccctttccagtatg3′; *ADIPSIN* UTR F: 5′tcaggagttcgagatcagca3′, R: 5′ctacaagcacccacctccat3′.

### Chromatin immunoprecipitation (ChIP)

Cultured human BMSC were used for ChIP using the Magna ChIP kit (Millipore Corporation, Billerica, MA) according to manufacturers’ instructions using 2 μg of TET1 (MABE 1034; Millipore Corporation) or 1 μg of TET2 (MABE 462) antibody. Purified DNA was then analysed by qPCR as previously described [33].

### hMeDIP assay

Human BMSC were cultured in normal growth media or in osteogenic inductive media for 1 week. Genomic DNA was prepared using the Qiagen genomic DNA isolation kit and then sonicated to generate 200–500 bp fragments. DNA was denatured in 10 mM Tris–HCl, 1 mM EDTA, pH 7.5 (TE buffer), for 10 min at 98 °C and then chilled on ice. 1% of denatured DNA was retained as the input control. Denatured 5hmC DNA was immunoprecipitated for 2 h at 4 °C with 2 μg of monoclonal antibody against 5hmC (Ab106918; Abcam, Cambridge, UK) in a final volume of 500 μl IP buffer (10 mM sodium phosphate (pH 7.0), 140 mM NaCl, 0.05% Triton X-100). The immunoprecipitation was incubated with magnetic IgG beads for 2 h at 4 °C and washed with IP buffer and then in high salt buffer (10 mM sodium phosphate (pH 7.0), 400 mM NaCl, 0.05% Triton X-100). Immunoprecipitated bead–DNA complex was treated with proteinase K for 3 h at 50 °C in elution buffer and hydroxymethylated DNA purified by using the Qiagen PCR clean-up kit (Qiaquick). DNA was quantitated using qPCR.

### Global 5hmc and 5mc analysis

The levels of 5mc and 5hmc were assayed by ELISA using either the Quest 5-hmc DNA ELISA Kit (Cat# D5425, Zymo Research) or 5-mc DNA ELISA Kit (cat# D5325, Zymo Research), respectively. Briefly, 96 well plates were coated with either anti-5-hydroxymethylcytosine polyclonal antibody or anti-5-methylcytosine polyclonal antibody (Zymo Research) (1 ng/μl) in coating buffer. BMSC were cultured for 2 weeks under normal, osteogenic or adipogenic inductive conditions. 100 ng of denatured DNA and standard DNA with known 5hmc or 5mc concentrations was added to the wells and incubated for 1 h. After washing, the cells were incubated with anti-DNA HRP conjugated antibody for 30 min. Wells were washed and HRP developer was added. The plates were read at 415 nm.

### Ovariectomised mouse model

Animal experiments and analyses were approved by the SA Pathology (23/11) and University of Adelaide (M-2013-144) Animal Ethics Committees. Twelve-week-old C57BL/6 female mice were anesthetised, and then their ovaries were either sited (sham surgery) or ovariectomised (OVX) as previously described [34]. The dorsal skin was then sutured and the mice were allowed to recover under normal housing conditions, with food and water provided ad libitum.

Three-dimensional micro-computed tomography (µCT) was performed as previously outlined [35] using Micro-CT (Skyscan 1076 X-ray Microtomography SkyScan, Bruker MicroCT, Kontich, Belgium, http://bruker-microct.com). Briefly, femora isolated from sham and OVX mice were scanned at 9 µm resolution, Al 0.5 mm filter, excitation 5890 ms, voltage 48 kV, current 110 mA, rotation step 0.6 and two-frame averaging. NRecon software was used to reconstruct the femora.

The incidence of murine clonogenic osteogenic progenitor cells was assessed using single-cell suspensions derived from bone marrow, and collagenase-treated crushed femoral bone chips were plated in colony-forming unit-fibroblast assays (CFU-F) which were performed as described previously [36].

Immunohistological staining was performed on sections (5 μm) of paraffin-embedded femora from OVX or sham surgery mice blocked using 5% horse serum (PIRL/SAHMRI; Gilles Plains, SA, Australia). Citrate antigen retrieval (DAKO; Glostrup, Denmark) was performed for 20 min at 95 °C, and endogenous peroxidase activity was quenched using 0.5% H_2_O_2_ in methanol for 40 min in the dark. IHC staining was performed using primary anti-5hmC antibody (Abcam; Cambridge, UK; 1/500 dilution), secondary biotinylated anti-rabbit IgG (Vector Labs; Burlingame, CA, USA; 1/250 dilution) and tertiary streptavidin conjugate (Life Technologies; Carlsbad, CA, USA; 1/100 dilution). Sections were stained using Liquid DAB^+^ Substrate Chromogen System (DAKO) and counterstained in Mayer’s haematoxylin.

### Statistics

Data analysis, graph generation and statistical analysis were carried out using Microsoft graphpad prism 6. Differentiation, ChIP assays and gene expression analysis were assessed by one-way ANOVA. Statistical differences (*) of *p* ≤ 0.05 between samples are shown.

## Results

### BMSC differentiation is dependent on DNA methylation

**Fig. 1:**
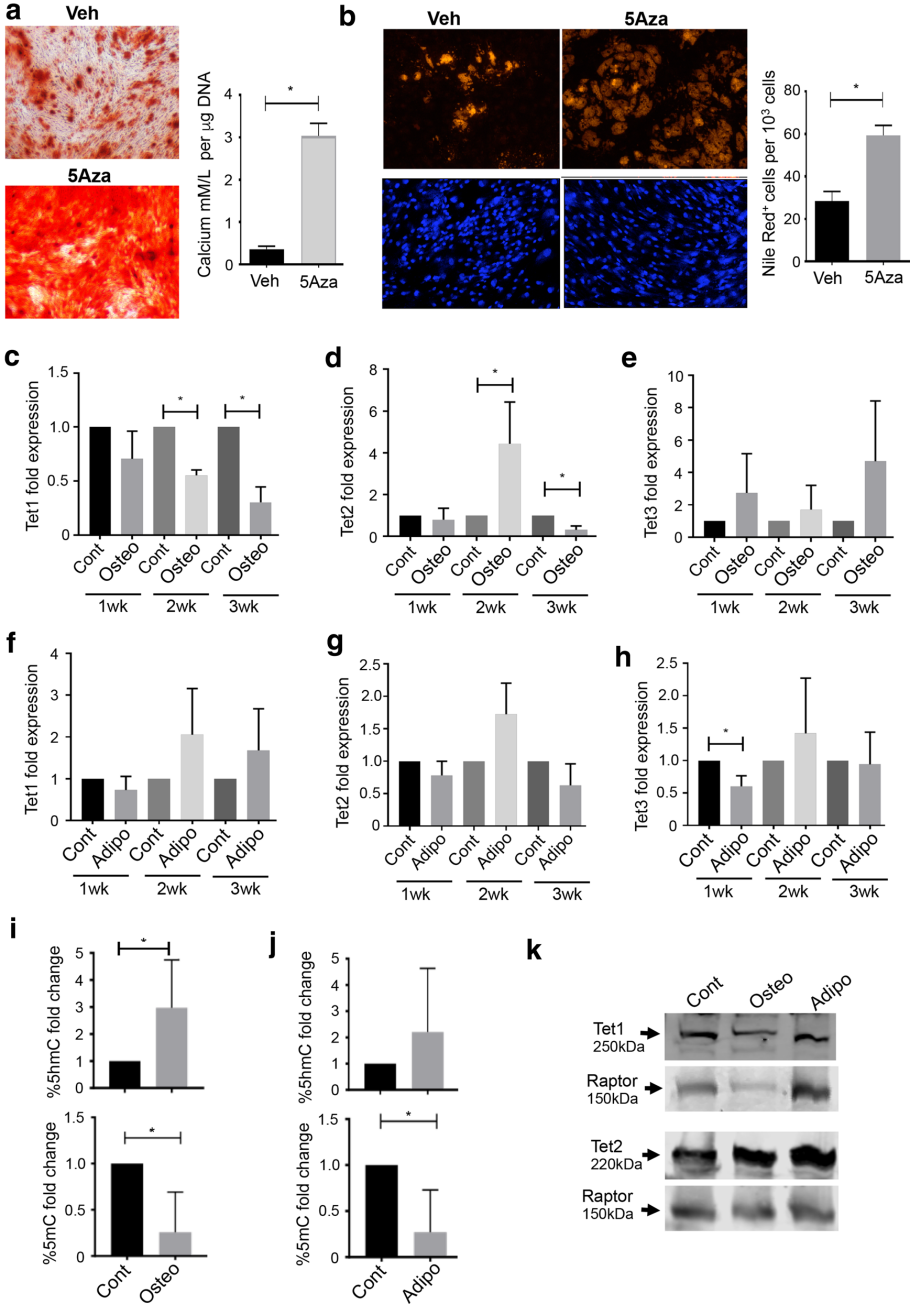
DNA demethylation promotes BMSC differentiation. **a** Cultured BMSC were treated with 2 μM 5-Azacytidine-2 (5-Aza2) or vehicle control (Veh) under osteogenic inductive conditions for 3 weeks. Mineralised deposits were stained with Alizarin red, and extracellular calcium levels were measured normalised to DNA content per well. **b** Cultured BMSC were treated with 5-Aza or Veh under adipogenic inductive conditions for 3 weeks. Lipid formation was assessed by Nile red staining, and the number of adipocytes was expressed as a percentage relative to total DAPI^+^ cells. Data represent *n* = 3 BMSC donors, *p < 0.05, Students *t* test. **c**–**e** BMSC cultured under osteogenic inductive (Osteo) or normal growth (Cont) conditions for 1 to 3 weeks and then analysed by qPCR to assess **c** *TET1*, **d** *TET2* and **e** *TET3* gene expression levels, relative to *β*-*actin*. **f**–**h** BMSC cultured under adipogenic inductive (Adipo) or Cont conditions for 1 to 3 weeks and then analysed by qPCR to assess **f** *TET1*, **g** *TET2* and **h** *TET3* gene expression levels relative to *β*-*actin.* Data represent mean S.E.M, *n* = 3 BMSC donors, *p < 0.05, one-way ANOVA with multiple comparison analyses. Human BMSC were cultured in Cont media or under Osteo or Adipo inductive conditions for 1 week. Genomic DNA was purified and global levels of **i** 5hmC and **j** 5mC were measured using by ELISA relative to total input DNA. Data represent *n* = 3 BMSC donors, *p < 0.05, Students *t* test. **k** Human BMSC were cultured under Cont, Osteo or Adipo inductive conditions for 2 weeks and then analysed by Western blot to assess TET1 and TET2 protein levels compared to Raptor as loading control

DNA methylation has been shown to regulate differentiation in many cell types including BMSC. Our initial studies attempted to verify the repressive function of DNA methylation in BMSC lineage determination. Human BMSC were cultured in the presence of the DNA methyl transferase inhibitor, 5 azacytidine (5Aza-2). Concentrations of 5Aza-2 greater than 2 μM were found to inhibit proliferation and induce cell death, and hence all functional experiments were performed with 2 μM 5Aza-2 (unpublished observations). BMSC cultured in the presence of 5Aza-2 under osteogenic inductive conditions produced larger aggregates of Alizarin red stained mineralised deposits, when compared to BMSC treated with vehicle alone (Fig. 1a). Moreover, 5Aza-2-treated BMSC exhibited significantly higher levels of extracellular calcium when normalised to DNA content per well, compared to vehicle-treated cells. Parallel studies found that BMSC treated with 5Aza2 under adipogenic inductive conditions resulted in increased numbers of Nile red-positive lipid-forming adipocytes compared to BMSC treated with the vehicle control (Fig. 1b). These results confirm that DNA methylation is inhibitory to BMSC differentiation.

### The role of hydroxymethylation during BMSC differentiation

Since DNA methylation is inhibitory to BMSC development and the enzymatic activity of TET family proteins results in the hydroxylation of methylated DNA (5mC) to 5hmC, which opposes the repressive functions associated with 5mC, we investigated whether the DNA hydroxylase TET family of enzymes regulate differentiation of BMSC. Analysis of *TET1*, *TET2* and *TET3* gene expression during BMSC differentiation showed that *TET1* transcript levels were significantly decreased at 2 and 3 weeks post-osteogenic induction, when compared to BMSC cultured under normal growth conditions (Fig. 1c). In contrast, *TET2* transcript levels were significantly increased at 2 weeks post-osteogenic induction (Fig. 1d) and then transcript levels decreased. *TET3* expression levels only showed a significant increase at 4 weeks of osteogenic induction compared to control-treated cells (Fig. 1e). Gene expression levels measured during adipogenic differentiation demonstrated that *TET1* transcript levels did not significantly change compared to BMSC cultured under normal growth conditions (Fig. 1f). Levels of *TET2* transcript did not show any significant changes during adipogenesis (Fig. 1g), whereas *TET3* showed a significant decrease at 1-week post-adipogenic induction (Fig. 1h). These data suggest that TET molecules may play a role in BMSC cell fate determination with more dynamic changes in gene expression evident during osteogenesis.

Given that 5hmc is an intermediate that leads to DNA demethylation and that we saw effects on BMSC differentiation when using the DNA methyltransferase inhibitor, we next assessed global levels of 5mc and 5hmc following osteogenic and adipogenic induction. The data showed a significant increase in 5hmc levels under osteogenic conditions for 1 week, with an associated downregulation of 5mc levels (Fig. 1i). Similarly, global 5hmc levels appeared elevated correlating with decreased 5mc levels post-1 week under adipogenic inductive conditions (Fig. 1j). Western blot analysis revealed that TET1 protein levels were relatively unchanged during BMSC differentiation, while TET2 protein levels were elevated following 2 weeks of osteogenic or adipogenic inductive conditions (Fig. 1k).

**Fig. 2:**
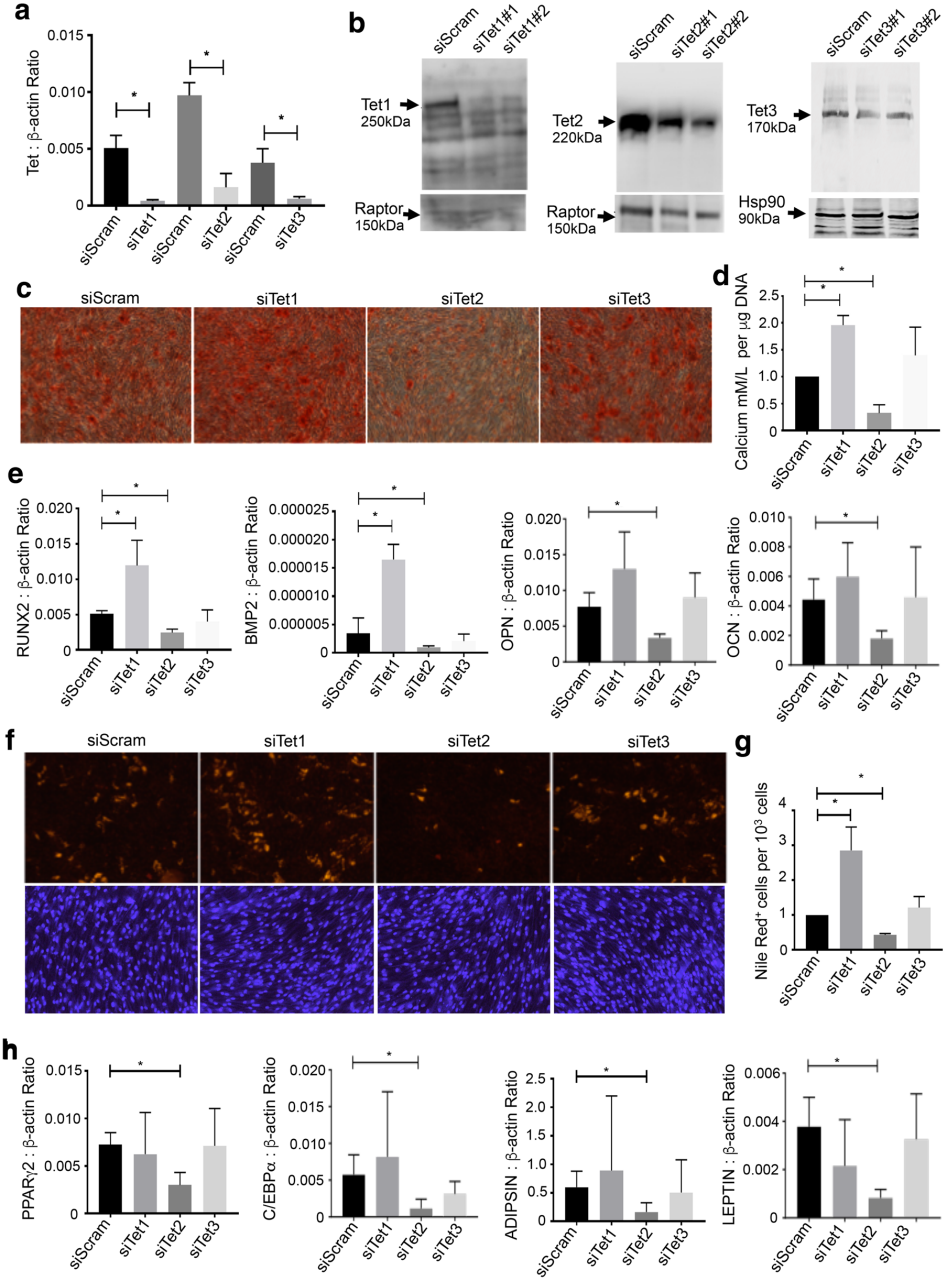
*TET1* and *TET2* knockdown differentially regulated BMSC differentiation. Cultured BMSC were transfected with siRNA targeting either *TET1* (siTET1), *TET2* (siTET2), *TET3* (siTET3) or scramble control siRNA (siScram). **a** Knockdown efficiency was analysed by qPCR relative to β-actin. **b** Knockdown of TET1, TET2 and TET3 protein levels by two siRNA per molecule was analysed by Western blot, using Raptor and Hsp90 protein as loading controls. **c** BMSC cultured under osteogenic inductive conditions were transfected with siTET1, siTET2, siTET3 or siScram. Representative images depict levels of Alizarin red-stained mineral deposits. **d** Extracellular calcium levels were measured and then normalised to DNA content per well. **e** Analysis of *RUNX2*, *BMP2, OSTEOPONTIN (OPN) and OSTEOCALCIN (OCN)* gene expression levels was assessed by qPCR per condition, relative to *β*-*actin*. **f** BMSC cultured under adipogenic inductive conditions were transfected with siTET1, siTET2, siTET3 or siScram. Representative images depict levels of Nile red-stained lipid droplets. **g** The number of adipocytes was expressed as a percentage relative to total DAPI-positive cells per unit area. **h** Analysis of *PPARγ2, C/EBPα, ADIPSIN* and *LEPTIN* gene expression levels was assessed by qPCR per condition, relative to *β*-*actin*. Data represent mean S.E.M., *n* = 3 BMSC donors, *p ≤ 0.05 One-way ANOVA with multiple comparison analyses

To address the potential role of TET molecules during BMSC differentiation, human BMSC were treated with either scramble control siRNA or siRNA specific to *TET1*, *TET2* or *TET3*. The gene expression levels of all three *TET* molecules were significantly knocked down compared to scrambled controls (Fig. 2a). These results were confirmed with two siRNAs directed to either *TET1, 2 or 3* (data not shown). Western blot analysis confirmed diminished TET protein levels following siRNA-mediated knockdown of *TET1, TET2* and *TET3* (Fig. 2b).

Functional studies showed that siRNA *TET1*-treated BMSC exhibited an enhanced osteogenic potential, compared to scramble siRNA-treated cells (Fig. 2c), whereas siRNA knockdown of *TET2* resulted in a significantly reduced osteogenic potential, as assessed by Alizarin red staining (Fig. 2c) and quantitation of extracellular calcium levels (Fig. 2d). However, siRNA knockdown of *TET3* showed no significant difference in mineral formation compared to scramble siRNA control cells (Fig. 2c, d). Supportive studies found that siRNA *TET1*-treated BMSC expressed significantly higher transcript levels of the osteogenic genes *RUNX2*, *BMP*-2, but not *OSTEOPONTIN* and *OSTEOCALCIN* compared to scramble siRNA-treated cells, whereas siRNA *TET2*-treated cells displayed a significant reduction in *RUNX2*, *BMP*-2, *OSTEOPONTIN* and *OSTEOCALCIN* gene expression levels (Fig. 2e).

Assessment of the role of TET proteins during BMSC adipogenic differentiation showed that siRNA knockdown of *TET1* resulted in an increase in Nile red-positive adipocytes, compared to the scramble siRNA control (Fig. 2f, g). Conversely, siRNA knockdown of *TET2* resulted in a substantial decrease in Nile red-positive adipocytes compared to the scramble siRNA-treated cells (Fig. 2f, g). However, siRNA knockdown of *TET3* showed no significant effect when compared to scramble siRNA-treated BMSC. Gene expression studies found that siRNA *TET2-*treated BMSC expressed significantly lower transcript levels of the adipogenic genes *PPARγ*2, C/EBPα, *ADIPSIN* and *LEPTIN* compared to scramble siRNA-treated cells (Fig. 2h) with no significant effect seen when TET1 or TET3 was knocked down.

**Fig. 3:**
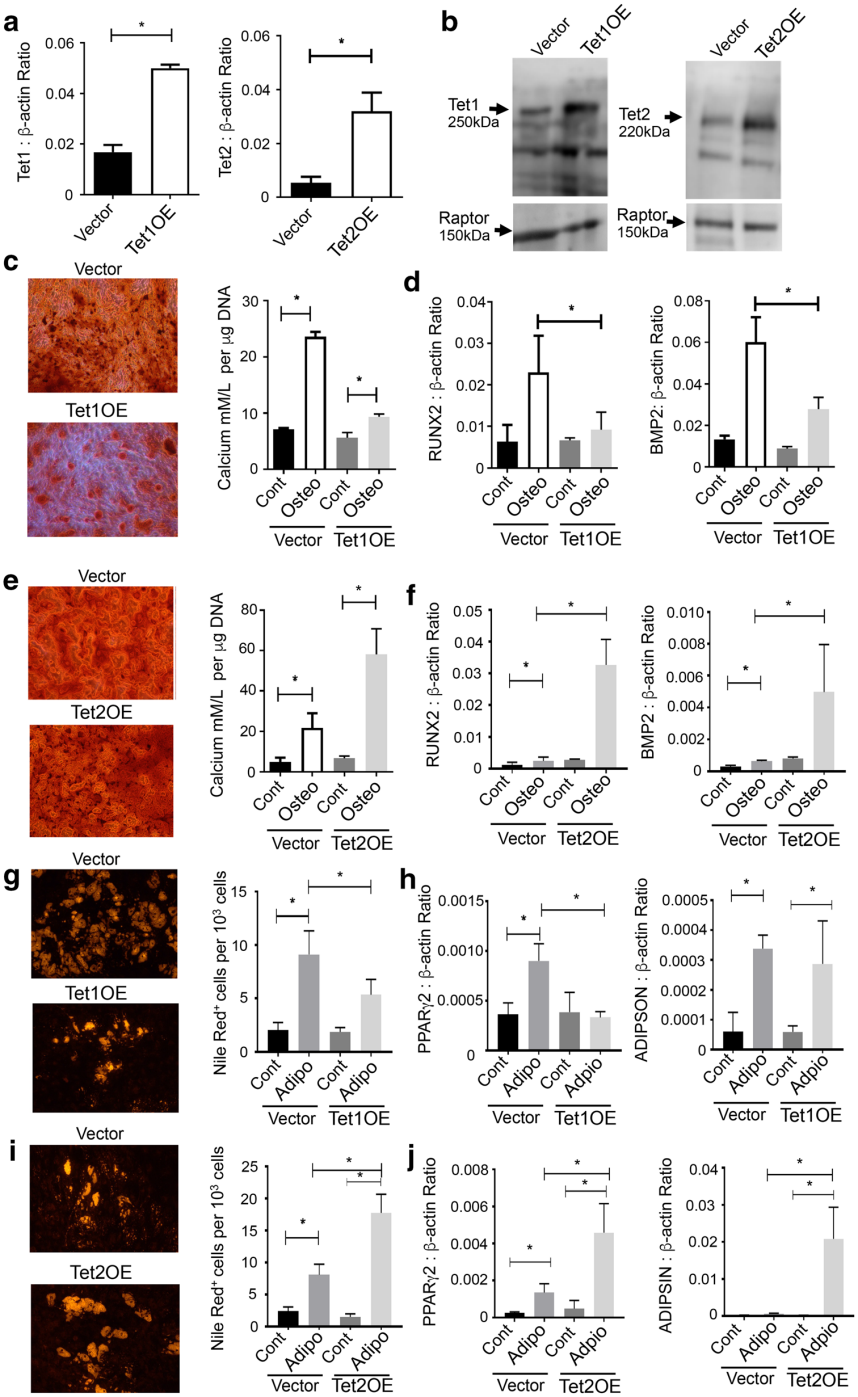
Overexpression of *TET1* and *TET2* differentially regulates osteogenesis and adipogenesis. **a** BMSC donors were infected with empty lentiviral vector alone (Vector), or vector overexpressing *TET1* cDNA (TET1OE) or *TET2* cDNA (TET2OE). Gene expression levels were analysed by qPCR relative to *β*-*actin*. **b** Protein levels of TET1 and TET2 were analysed by Western blot, using Raptor protein as a loading control. **c** TET1-overexpressing BMSC were cultured under osteogenic (Osteo) inductive conditions control media (Cont) for 3 weeks. Representative images depict mineralised deposits stained using Alizarin red. Extracellular calcium levels were measured and then normalised to DNA content per well. **d** Analysis of *RUNX2* and *BMP2* gene expression levels was assessed by qPCR per condition, relative to *β*-*actin*. **e** TET2-overexpressing BMSC were cultured under Osteo or Cont media for 3 weeks. Representative images depict mineralised deposits stained with Alizarin red. Extracellular calcium levels were normalised to DNA content per well. **f** Analysis of *RUNX2* and *BMP2* gene expression levels was assessed by qPCR per condition, relative to *β*-*actin*. **g** TET1-overexpressing BMSC were cultured under adipogenic (Adipo) inductive conditions or Cont media for 3 weeks. Representative images depict levels of Nile red-stained lipid droplets. The number of adipocytes was expressed as a percentage relative to total DAPI positive cells per unit area. **h** Analysis of *PPARγ2* and *ADIPSIN* gene expression levels was assessed by qPCR per condition, relative to *β*-*actin*. **i** TET2-overexpressing BMSC were cultured under Adipo or Cont media for 3 weeks. Representative images depict levels of Nile red-stained lipid droplets. The number of adipocytes was expressed as a percentage relative to total DAPI-positive cells per unit area. **j** Analysis of *PPARγ2* and *ADIPSIN* gene expression levels was assessed by qPCR per condition, relative to *β*-*actin*. Data represent mean S.E.M., *n* = 3 BMSC donors, *p ≤ 0.05, one-way ANOVA with multiple comparison analyses

The function of TET1 and TET2 during BMSC differentiation was confirmed by enforced expression of either *TET1* or *TET2* full-length cDNA in BMSC and compared to empty vector controls. Lentiviral-mediated overexpression of *TET1* and *TET2* was verified using qPCR (Fig. 3a) and Western blot analyses (Fig. 3b). Stably transduced BMSC were subsequently cultured under osteogenic inductive conditions and assessed for mineral formation. *TET1*-overexpressing BMSC produced lower amounts of Alizarin red mineralised deposits compared to empty vector control BMSC, correlating with significantly lower extracellular calcium levels (Fig. 3c). Gene expression studies found that *TET1*-overexpressing BMSC exhibited lower transcript levels of *RUNX2* and *BMP*-2 under osteogenic inductive conditions compared to vector control BMSC (Fig. 3d). In contrast, *TET2-*overexpressing BMSC exhibited greater amounts of Alizarin red stained mineralised deposits, correlating with significantly higher levels of extracellular calcium levels (Fig. 3e). Confirmatory qPCR showed that *TET2-*overexpressing BMSC expressed significantly higher levels of *RUNX2* and *BMP*-2 transcripts under osteogenic inductive conditions compared to vector control BMSC (Fig. 3f).

We next sought to determine the contribution of both TET1 and TET2 to global 5hmC and 5mC demethylation. Human BMSC were transfected with either siRNA targeting *TET1* or *TET2* alone or both *TET1/TET2* in combination and then cultured under control or osteogenic inductive media. The data showed significant effects on global 5hmc levels only when both *TET1* and *TET2* were knocked down in combination under normal and osteogenic conditions (Additional file 1: Figure S1a). Examination of 5mC revealed that dual knockdown of both *TET1* and *TET2* caused significantly higher levels of global 5mC under both normal and osteogenic conditions (Additional file 1: Figure S1a). Functional analyses found that osteogenesis was impeded in the presence of siRNA targeting *TET2,* negating the increase in BMSC mineralisation following treatment with si*TET1* (Additional file 1: Figure S1c). Therefore, both TET1 and TET2 appear to contribute to global 5hmC and 5mC demethylation but work through different mechanisms to regulate BMSC osteogenic differentiation.

Under adipogenic inductive conditions, overexpression of *TET1* inhibited the amount of Nile red-positive, lipid-containing adipocytes compared to BMSC infected with empty vector (Fig. 3g). Gene expression analyses demonstrated significantly lower levels of *PPARγ*2 and transcript in *TET1*-overexpressing BMSC cultured under adipogenic inductive conditions compared to vector control BMSC (Fig. 3h). Conversely, overexpression of *TET2* promoted higher numbers of lipid-forming adipocytes compared to vector control BMSC (Fig. 3i). Supportive studies showed that *TET2*-overexpressing BMSC exhibited significantly higher levels of *PPARγ*2 and *ADIPSIN* under adipogenic inductive conditions, compared to vector control BMSC (Fig. 3j). Collectively, these findings indicate that TET1 represses differentiation of BMSC into osteoblasts or adipocytes and that TET2 promotes BMSC differentiation.

### Deregulation of *TET1* and *TET2* expression and activity during osteoporosis

**Fig. 4:**
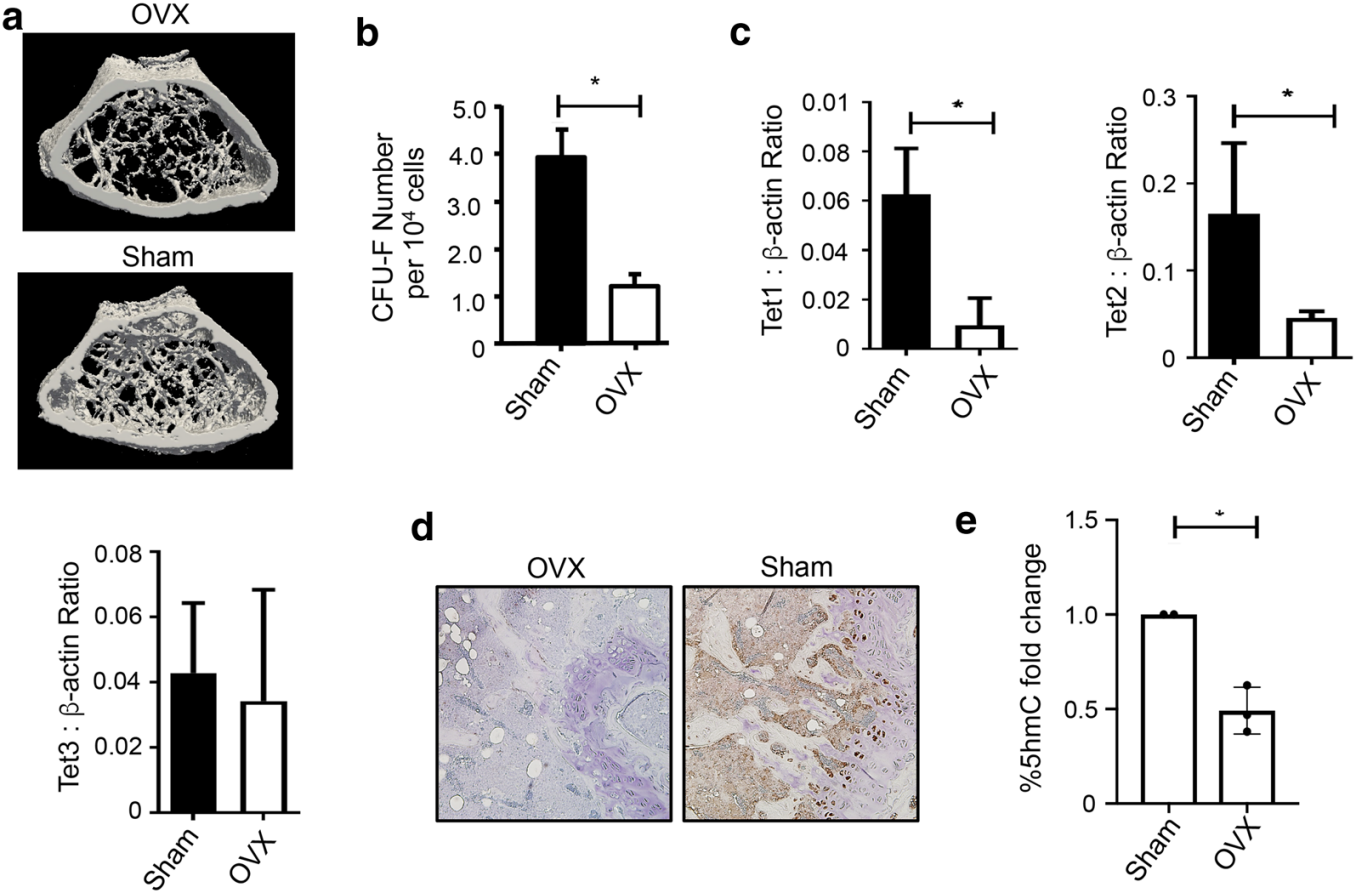
Osteoprogenitor cell numbers and *TET1/2* gene expression are reduced in osteoporotic bones. Ovariectomy (OVX) or sham surgeries (Sham) were performed on 12-week-old C57/BL6 female mice. **a** Trabeculae in femora harvested 12 weeks post-OVX or Sham treatment analysed by μCT. **b** The number of clonogenic stromal cells (CFU-F) was assessed from bone marrow and crushed femoral bone isolated from OVX or Sham mice, 12 weeks post-treatment. **c** Gene expression levels of *TET1, TET2 and TET3* normalised to *β*-*actin* were measured by qPCR, in stromal cells isolated from the femora of OVX or Sham mice, 12 weeks post-treatment. **d** Immunohistochemical analysis of 5 μM tibial sections stained with anti-5hmC antibody, in OVX or Sham mice, 12 weeks post-treatment. 5hmC staining was detected on bone surfaces in primary spongiosa and chondrocytes in growth plate region. **e** Cultured stromal cells derived from the femora of OVX mice and Sham control mice were cultured in normal media, genomic DNA was purified and global levels of 5hmC and 5mC were measured by ELISA relative to total input DNA. Data represent mean S.E.M., *n* = 5 mice per condition donors, *p ≤ 0.05, two-tailed Student *t* test

During the process of skeletal aging and the development of diseases such as age-related osteoporosis, BMSC numbers and differentiation potential can be compromised, leading to increased adipogenesis in the bone marrow at the expense of bone formation [37]. As our results clearly show a function for TET molecules in BMSC lineage determination, we performed experiments to assess the levels of TET genes and 5hmC during osteoporosis. Femora harvested from mice 12-week post-ovariectomy showed reduced levels of trabeculae bone and per cent bone relative to tissue volume by μCT analysis, compared to aged matched sham surgery controls (Fig. 4a). These data correlated with lower numbers of colony-forming units-fibroblastic (CFU-F) in overiectomised mice compared to sham controls (Fig. 4b). Gene expression studies of freshly isolated stromal cells (Sca1+/Lin−/CD31−/CD45−) demonstrated that transcript levels for both *TET1* and *TET2* were significantly reduced in ovariectomised mice, whereas *TET3* expression showed no significant difference, when compared to sham controls (Fig. 4c). In support of the reduced levels of *TET1* and *TET2*, we also observed a reduced global staining of 5hmc in femoral sections derived from overiectomised mice compared to sham-treated mice (Fig. 4d). Confirmatory analyses showed significantly higher levels of global 5hmC in cultured stromal cells derived from OVX mouse limb bone compared to sham surgery control mouse bone (Fig. 4e).

### TET1 and TET2 are responsible for 5hmC on lineage genes

**Fig. 5:**
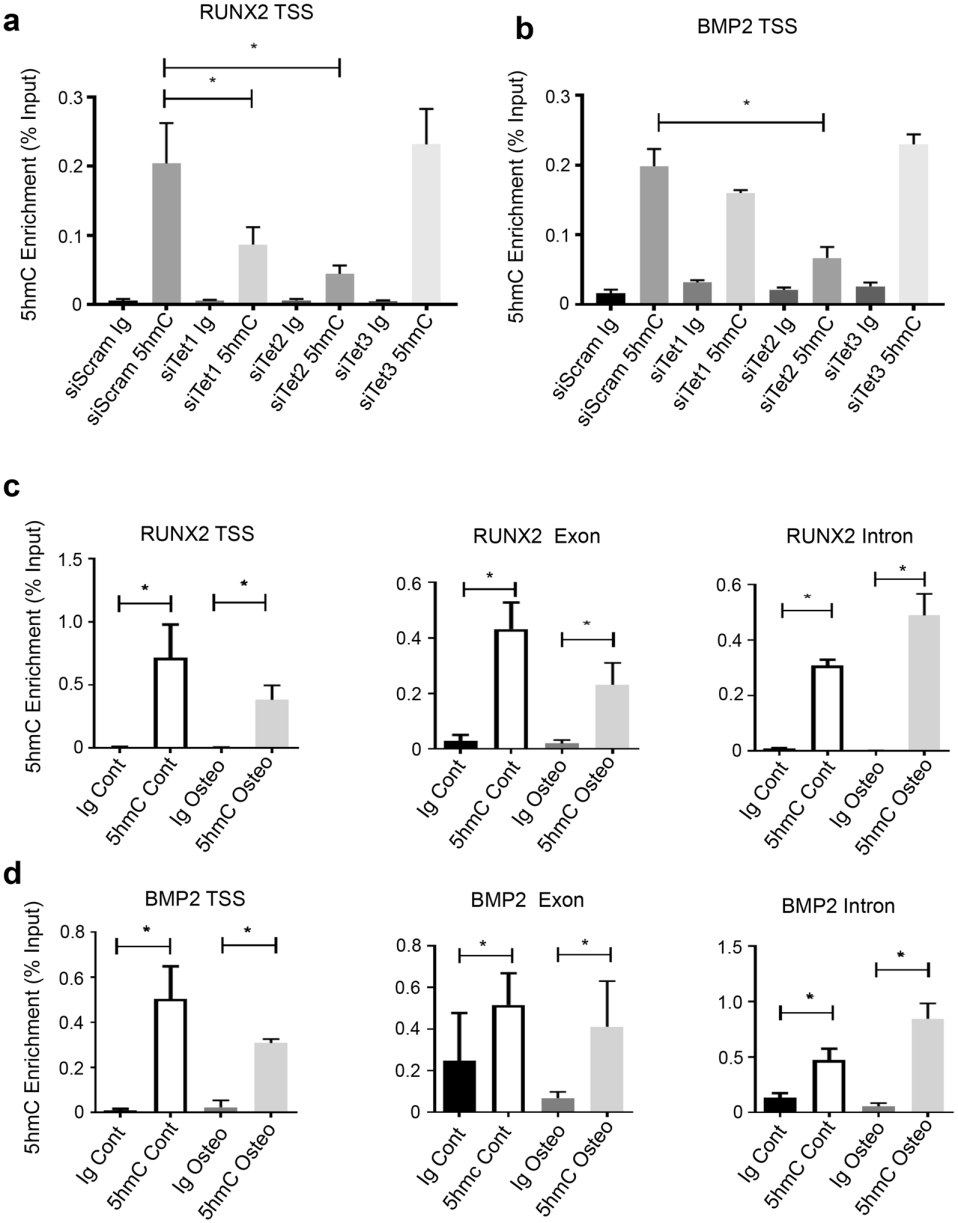
TET1 and TET2 influence 5hmC on osteogenic genes. BMSC were cultured under normal growth conditions and treated with scramble siRNA or siRNA directed to *TET1* (siTET1) or *TET2* (siTET2) and genomic DNA purified and immunoprecipitated using an antibody to 5hmC. Recruitment of 5hmC to genomic regions was assessed by the hme-DIP analysis and normalised to the genomic input control. **a** Relative enrichment of 5hmC on *RUNX2* transcription start site (TSS) was measured using PCR. **b** 5hmC on *BMP2* TSS was measured as in (**a**). BMSC were cultured under normal growth and genomic DNA purified and immunoprecipitated using an antibody to 5hmC. Recruitment of 5hmc to genomic regions was assessed by the hme-DIP analysis and normalised to the genomic input control. **c** Cells were cultured under normal and osteogenic conditions. Relative enrichment of 5hmC on *RUNX2* transcription start site (TSS), exon and intron regions. **d** Relative enrichment of 5hmC on *BMP2* TSS, exon and intron regions, percentage input. Data represent mean S.E.M., *n* = 3 BMSC donors, *p ≤ 0.05, one-way ANOVA with multiple comparison analyses

Given that the TET1 and TET2 enzymes convert 5mC on DNA to 5hmC, h-MeDIP analysis was employed to determine the 5hmC status of the lineage markers. To determine which TET member is responsible for 5hmC on lineage genes, we knocked down all three TET family members individually and assessed 5hmC on the TSS under normal culture conditions. Knockdown of both *TET1* and *TET2* resulted in a significant decrease in 5hmC along the RUNX2 TSS, whereas knockdown of *TET3* had no significant effect (Fig. 5a). Knockdown of *TET2* significantly reduced 5hmC on the *BMP2* TSS but knockdown of *TET1* or *TET3* had no significant effect (Fig. 5b). We next examined whether 5hmc levels change during osteogenic differentiation. The data demonstrated significant levels of 5hmC on the *RUNX2* TSS, exon and intron, when compared to the IgG control in both control and osteogenic inductive conditions (Fig. 5c). The same was evident for the *BMP2* TSS, exon and intron (Fig. 5d).

**Fig. 6:**
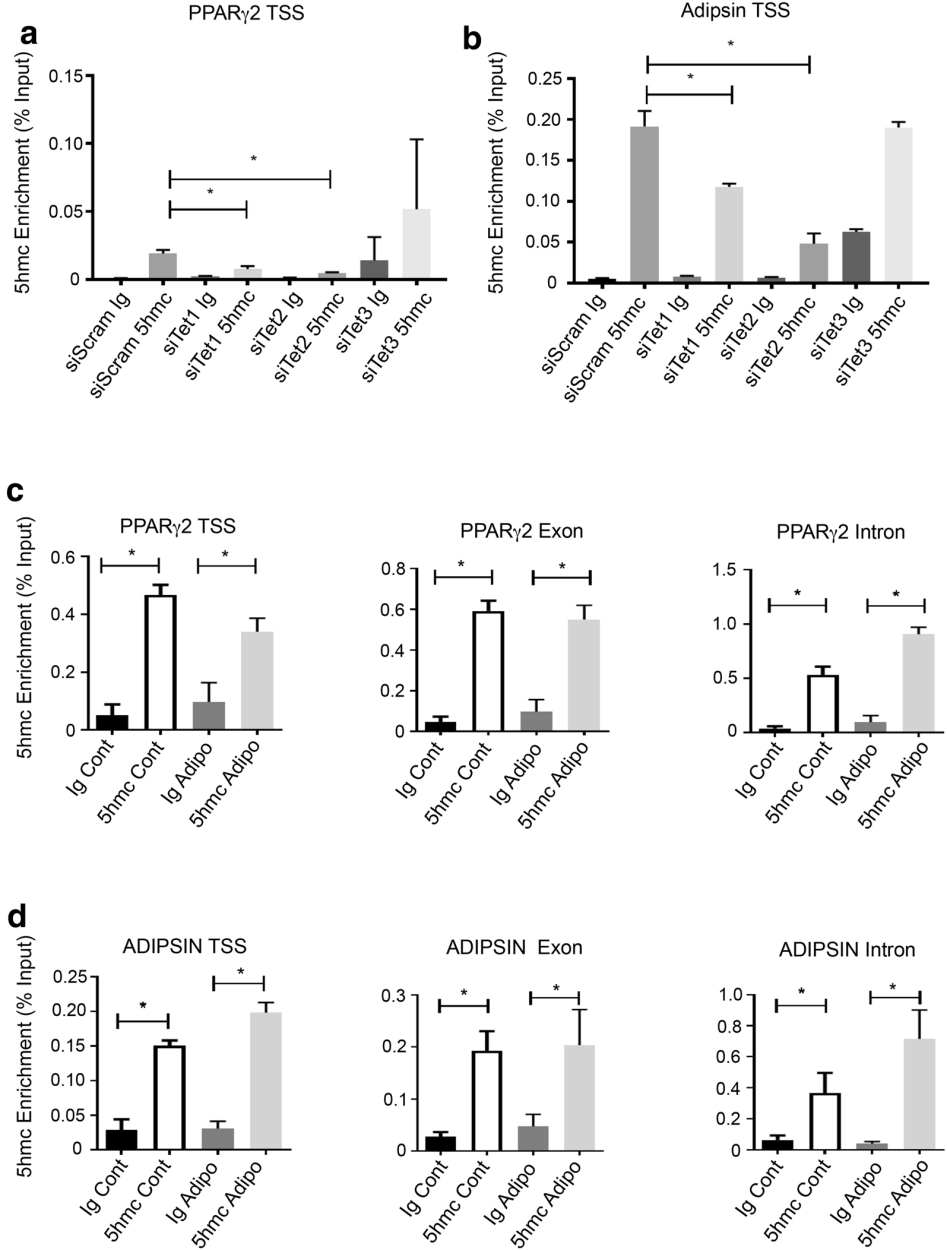
TET1 and TET2 influence 5hmC on adipogenic genes. BMSC were cultured under normal growth conditions and treated with scramble siRNA or siRNA directed to *TET1* (siTET1) or *TET2* (siTET2) and genomic DNA purified and immunoprecipitated using an antibody to 5hmC. Recruitment of 5hmC to genomic regions was assessed by the hme-DIP analysis and normalised to the genomic input control. **a** Relative enrichment of 5hmC on *PPARγ* transcription start site (TSS) and **b** *ADIPSIN* was measured using qPCR. **c** BMSC were cultured under normal growth (Cont) or adipogenic (Adipo) conditions and then processed for chromatin extraction. Relative enrichment of 5hmC on *PPARγ2* transcription start site (TSS), exon and intron regions. **d** Relative enrichment of 5hmC on *ADIPSIN* TSS, exon and intron regions. Data represent mean S.E.M., *n* = 3 BMSC donors, *p ≤ 0.05, one-way ANOVA with multiple comparison analyses

We next examined 5hmc on adipogenic genes. siRNA knockdown of *TET1* or *TET2* resulted in a significant decrease in 5hmC for both *PPARγ2* and *ADIPSIN,* whereas *TET3* had no effect (Fig. 6a, b).We then examined levels of 5hmc during adipogenic differentiation. 5hmc was present on the *PPARγ2* TSS, exon and intron (Fig. 6c), where levels remained constant under adipogenic conditions. Similar findings were evident for the *ADIPSIN* TSS, exon and intron (Fig. 6d).

### TET1 and TET2 directly bind lineage genes

**Fig. 7:**
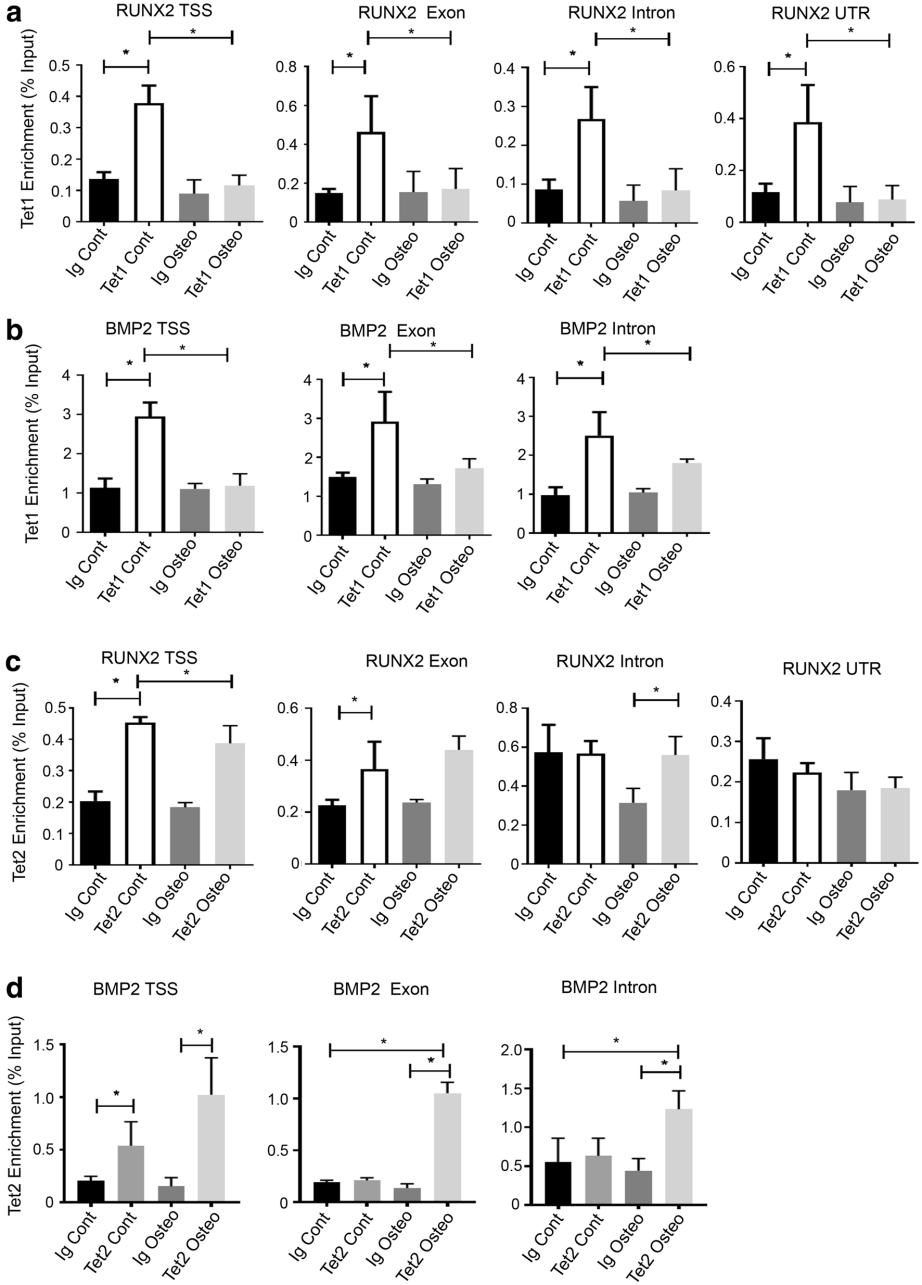
TET1 and TET2 differentially bind to osteogenic gene regions. BMSC were cultured under normal growth (Cont) or osteogenic (Osteo) conditions and then processed for chromatin extraction. Recruitment of TET molecules to genomic regions was assessed by chromatin immunoprecipitation and normalised to the genomic input control. **a** Relative enrichment of TET1 on *RUNX2* transcription start site (TSS), exon, intron and 3′ untranslated region (UTR). **b** Relative enrichment of TET1 on *BMP2* TSS, exon and intron regions. **c** Relative enrichment of TET2 on *RUNX2* TSS, exon, intron and UTR. **d** Relative enrichment of TET2 on *BMP2* TSS, exon and intron regions. Data represent mean S.E.M., *n* = 3 BMSC donors, *p ≤ 0.05, one-way ANOVA with multiple comparison analyses

ChIP analysis was employed to examine whether TET1 and TET2 directly bind to lineage-specific genes to regulate transcription. Assessment of the level of TET1 occupancy along the *RUNX2* gene locus found that TET1 was enriched on the *RUNX2* transcription start site (TSS) (Fig. 7a), with similar enrichment along the exon, intron and 3′UTR, under normal growth conditions compared to Ig control. However, under osteogenic inductive conditions, the levels of TET1 on the *RUNX2* gene locus decreased dramatically. Significant levels of TET1 were also present along the *BMP2* TSS (Fig. 7b), exon and intron, under normal growth conditions; however, TET1 binding levels were significantly decreased during osteogenesis. Assessment of TET2 occupancy by ChIP analysis found significant levels of enrichment along the *RUNX2* TSS (Fig. 7c), exon but not the intron or 3′UTR under normal growth conditions compared to the IgG control. During osteogenesis, TET2 levels remained the same along the *RUNX2* TSS (Fig. 7c) and exon but increased along the intron, with no significant changes on the UTR. TET2 was enriched along the *BMP2* TSS under control conditions (Fig. 7d), and along the TSS, exon and intron of *BMP2* under osteogenic conditions.

**Fig. 8:**
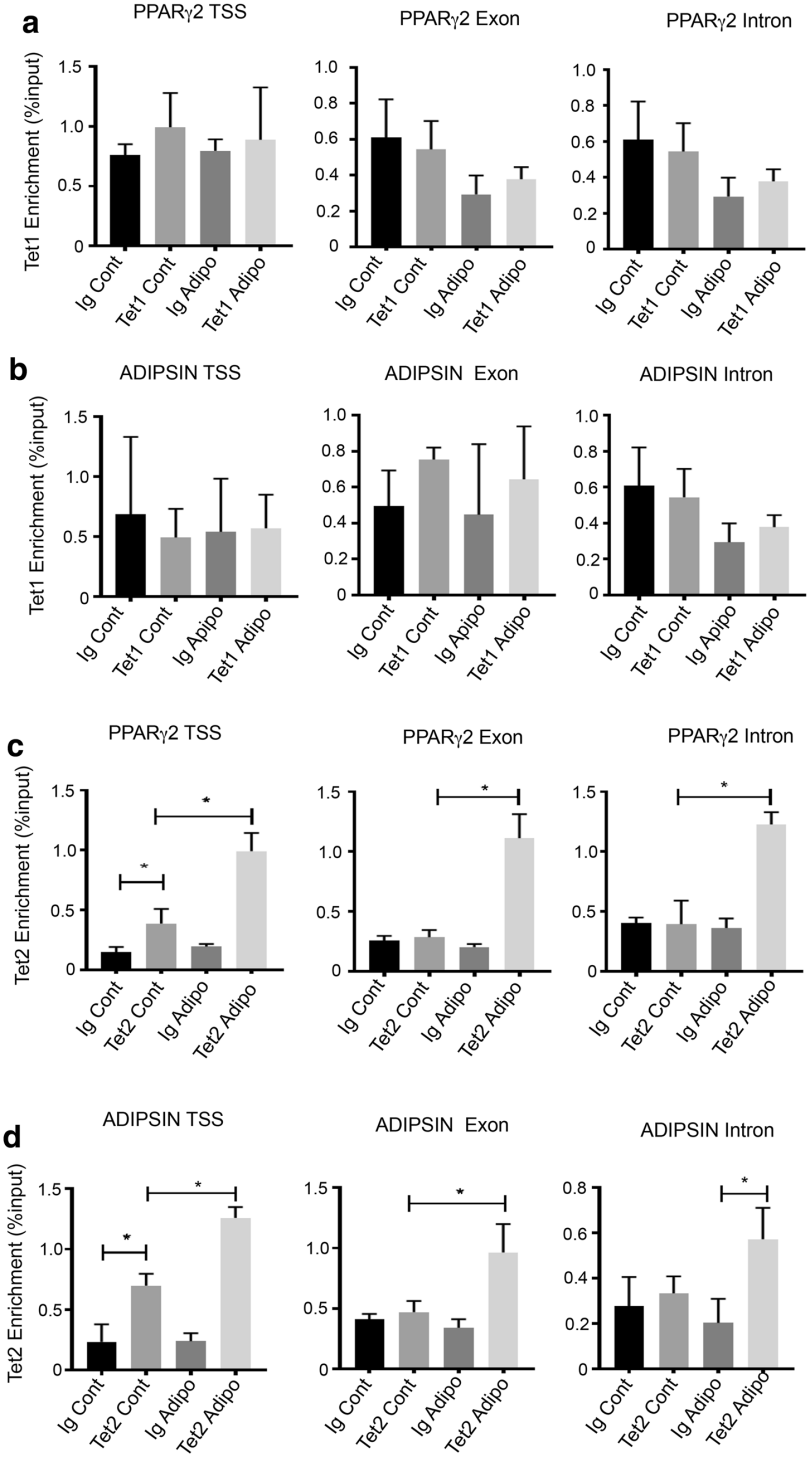
TET1 and TET2 differentially bind to adipogenic gene regions. BMSC were cultured under normal growth (Cont) or adipogenic (Adipo) conditions and then processed for chromatin extraction. Recruitment of TET molecules to genomic regions was assessed by chromatin immunoprecipitation and normalised to the genomic input control. **a** Relative enrichment of TET1 on *PPARγ2* transcription start site (TSS), exon and intron regions. **b** Relative enrichment of TET1 on *ADIPSIN* TSS, exon and intron regions. **c** Relative enrichment of TET2 on *PPARγ2* TSS, exon and intron regions. **d** Relative enrichment of TET2 on *ADIPSIN* TSS, exon and intron regions. Data represent mean S.E.M., *n* = 3 BMSC donors, *p ≤ 0.05, one-way ANOVA with multiple comparison analyses

Occupancy of TET1 and TET2 was also examined on adipogenic promoters. The data showed that TET1 was not enriched on *PPARγ2* or *ADIPSIN* genes under normal growth or adipogenic inductive conditions (Fig. 8a, b). However, TET2 was found to be recruited to *PPARγ2* TSS and *ADIPSIN* TSS under normal growth conditions but not the exons and introns. TET2 recruitment increased under adipogenic conditions and was present along exons and introns (Fig. 8c, d).

**Fig. 9:**
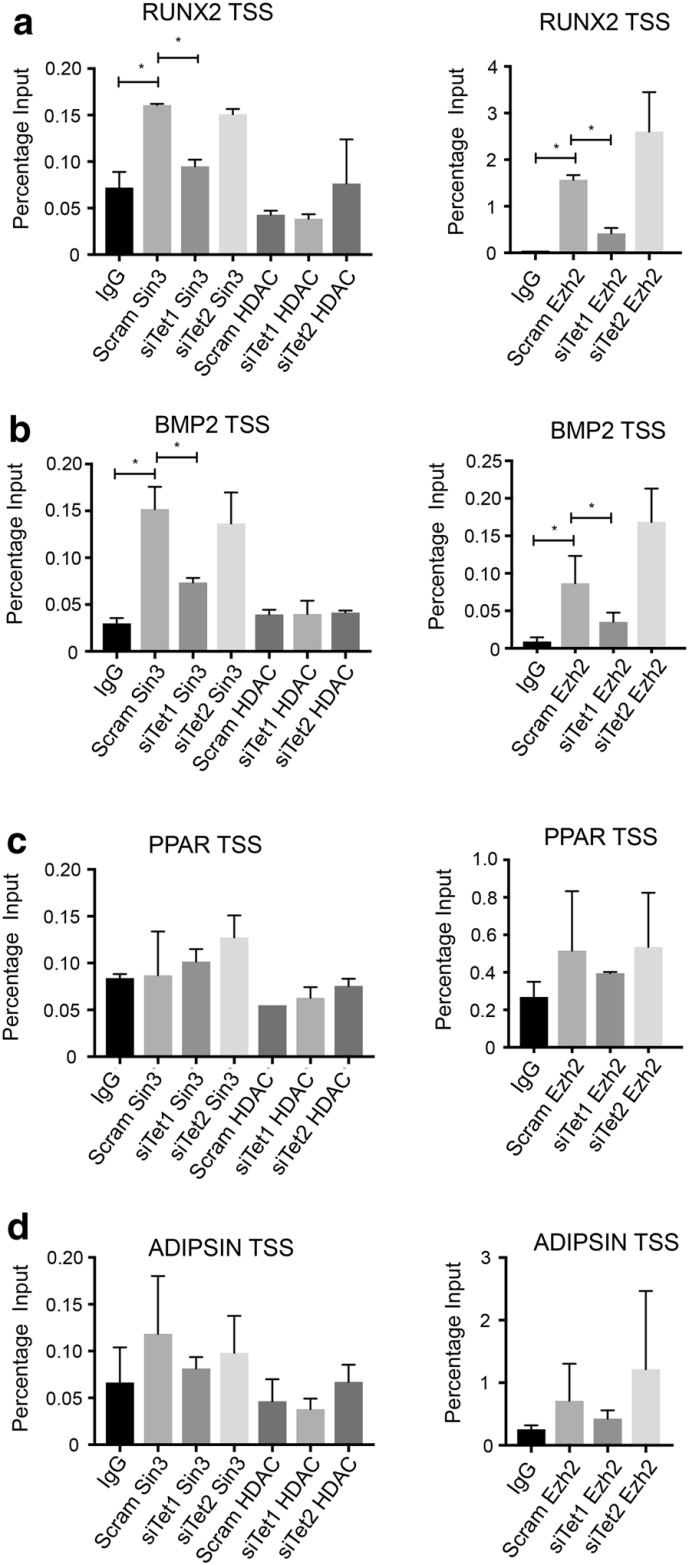
TET1 influences SIN3A and EZH2 binding to osteogenic genes. **a** BMSC were treated with scramble siRNA (scram), siTET1 or siTET2, cultured under normal growth and then processed for chromatin extraction. Recruitment of SIN3A, HDAC2 or EZH2 molecules to the *RUNX2* transcription start site (RUNX2 TSS) was assessed by chromatin immunoprecipitation and normalised to the genomic input control. **b** Cells were treated as in (**a**) but enrichment was assessed on the *BMP2* transcription start site (BMP2 TSS). **c** Cells were treated as in (**a**) but enrichment was assessed on the PPARγ transcription start site (PPAR TSS). **d** Cells were treated as in (**a**) but enrichment was assessed on the *ADIPSIN* transcription start site (ADIPSIN TSS). Data represent mean S.E.M., *n* = 3 BMSC donors, *p ≤ 0.05, one-way ANOVA with multiple comparison analyses

We have previously reported that the histone H3K27 methyl transferase, EZH2, is recruited to osteogenic genes, repressing osteogenic gene expression. TET1 has been shown to interact with SIN3A, HDAC1 and promote EZH2 recruitment to promoters. We therefore examined whether TET1 or TET2 can recruit these proteins in BMSC to lineage-specific promoters. Cells were treated with scramble siRNA or siRNA targeting TET1 or TET2 and assessed for recruitment of SIN3A, HDAC and EZH2. SIN3A was specifically recruited to the *RUNX2* TSS in scramble-treated cells, and specific recruitment was eliminated when *TET1* was knocked down but not when *TET2* was knocked down. No specific recruitment of HDAC was evident (Fig. 9a). EZH2 was recruited on the *RUNX2* TSS, which was reduced when *TET1* was knocked down but not when *TET2* was knocked down. A similar profile was evident for *BMP2*. SIN3A and EZH2 were recruited to the *BMP2* TSS. This was drastically reduced when *TET1* was knocked down but not *TET2* (Fig. 9b). Furthermore, we failed to observe any specific recruitment of SIN3A, HDAC or EZH2 on adipogenic genes, *PPARγ* and *ADIPSIN* (Fig. 9c, d).

## Discussion

In this study, we confirmed that inhibition of DNA methylation promotes human BMSC differentiation as treatment with 5-Aza2 showed increased osteogenesis and adipogenesis. This prompted us to examine the importance of 5hmc in BMSC differentiation, which has been shown to counteract the effects of DNA methylation by removing the methylation mark or inhibiting further methylation. Functional assessment of TET molecules found that TET1 inhibits both osteogenesis and adipogenesis by repressing transcription of *RUNX2* and *PPARγ2*, two essential master regulators of BMSC osteogenic and adipogenic commitment, respectively. Even though TET2 is the main enzyme responsible for 5hmC on osteogenic and adipogenic genes, we found that TET1 is also partly responsible for 5hmC directly on osteogenic and indirectly on adipogenic genes as we did not detect recruitment of TET1 on adipogenic genes. However, TET2 was recruited on the adipogenic genes; hence, it is likely that TET1 possibly is in a complex with TET2 or it influences the recruitment of TET2 to the adipogenic genes.

Functionally, however, TET1 represses transcription of osteogenic and adipogenic genes, which conflicts with its ability to influence 5hmC. Further analysis revealed that TET1 influences the recruitment of SIN3A and EZH2 to the osteogenic promoters. This was not evident for TET2. We attribute the repressive function of TET1 to its ability to promote recruitment of the repressors, SIN3A and EZH2, to osteogenic genes. We showed that TET1 and not TET2 is responsible for their recruitment. Previous studies have reported that TET1 can exist in a complex with Sin3A and HDAC, recruiting them to promoters to influence the recruitment of Ezh2 [28, 38–40]. This is consistent with our previous findings showing that Ezh2 is recruited to osteogenic genes and inhibits osteogenesis [21]. This finding therefore has provided some insight into how Ezh2 is recruited to the promoters of osteogenic genes.

We previously did not detect Ezh2 on adipogenic genes nor have we now found TET1 to be recruited. It is likely that TET1 is repressing an upstream activator of adipogenesis, and this will be the basis of future investigations. Secondly, despite the reduction in 5hmC when TET1 is knocked down, the increase in gene expression and osteogenic and adipogenic differentiation indicates that its repressive function is the most prominent feature in relation to differentiation. TET1 genomic location in ESC has been shown to overlap with PRC2, Ezh2, Sin3A and H3K27me3, where it can be recruited by the PRC2 complex to bivalent sites. TET1 has also been found to co-immunoprecipitate with PRC2 complex proteins, Ezh2 and SUZ12c. It was reported that with TET1-dependent demethylation and Ezh2-mediated repression through Ezh2, the TET1–PRC2 complex maintains developmental genes in a poised state of activation and is thought to contribute to plasticity [38]. Genome-wide studies show that TET1 has a preference to bind gene-rich regions with CpG-rich promoters with highest levels of recruitment along the transcription start sites and less so throughout the gene body [41]. A majority of TET1-bound promoters are also marked with H3K4me3, high CpG content which are hypomethylated in support of the role of TET1 in removing DNA methylation [42]. A second set of TET1-bound genes was associated with H3K27me3 and corresponded to the binding of the PRC2 complex [43]. A significant amount of these genes are involved in differentiation and development, with TET1 associated with H3K27me3 or bivalent domains. 5hmC is enriched at promoter regions and TSS and mainly on regions enriched in CpG islands, which are devoid of 5mC implying that TET1 via its ability to hydroxylate 5mC contributes to the hypomethylated signature of CpG islands in ESC [40].

In our study, TET2 was found to promote BMSC cell lineage commitment towards either an osteoblast or adipocyte fate, partly via transcriptional activation of *Runx2* or *PPARγ2,* respectively, and it was the main protein of the TET family responsible for 5hmC on both osteogenic and adipogenic genes. Interestingly, the third TET family member, TET3, appeared to have no effect on human BMSC differentiation. Our finding that TET1 is a potential inhibitor of human BMSC differentiation is in accord with previous studies identifying a functional role for TET1 in ESC, iPSC and neuronal stem cell development [44, 45]. TET1-deficient ESC display impaired self-renewal capacity, partly because of increased methylation and decreased expression of *Nanog* [5]. Another study, however, reported that *TET1*-deficient ESC displayed normal levels of pluripotency genes [46]; however, studies consistently show an increase in lineage-specific markers, such as trophoectoderm and neuroectoderm, associated with developmental skewing towards endoderm and mesoderm fates [44]. Triple knockout of TET1-3 in mESC shows results in an overactive Wnt signalling which results in skewed differentiation to mesoderm at the expense of neuroectoderm [47]. Wnt signalling is important in osteogenesis and adipogenesis, and it would be of interest to determine the effect of TET1–TET3 on Wnt signalling and differentiation in bone marrow-derived MSC. These findings correlate with our observations that reduction of *TET1* expression resulted in an increase in osteogenesis and adipogenesis, while overexpression of *TET1* inhibited BMSC differentiation.

In agreement, with our data a recent study found that TET2 is mainly responsible for inducing adipogenesis of a murine preadipocyte cell line 3T3-L1 and was responsible for 5hmC of the PPARγ locus [48]. In another study, TET2 and TET1 were found to promote osteoblast formation of murine mesenchymal multipotent C3H cells using short hairpin RNA. Both TET1 and TET2 were found to promote 5hmC [49]. TET2 has been found to be responsible for neurogenic differentiation of neuronal stem cells and is primarily responsible for the 5hmC dynamics [50]. Another study showed that knockdown of TET2 but not TET1 inhibited myogenic differentiation of myoblasts and was responsible for 5hmC [51]. In agreement, previous studies have found that TET2 is master regulator of smooth muscle cell differentiation [10].

In mESC, it has been shown that TET2 knockout results in 90% reduction in 5hmC, whereas knockout of TET1 results in 44% reduction. TET2^−/−^ mESC showed delayed gene transcription kinetics during ESC differentiation to the neuronal lineage [52]. Although the TET2 data are in agreement, the TET1 functional data contradict ours. This could be due to TET1 having a different function in the murine MSC cell line as opposed to primary human MSC. Another study has shown using a murine teratocarcinoma cell line, ATDC5, that TET1 promotes chondrogenesis and 5hmC by short hair pin RNA [53]. Similarly, TET1 knockdown using shRNA lentiviral vectors shows that TET1 promotes odontogenic differentiation of dental pulp stem cells [54]. A recent study by Yang et al. found that *TET1* and *TET2* deficiency in mouse bone cells reduced demethylation of the P2rX7 promoter and thus downregulated exosome release, leading to intracellular accumulation of miRNAs which inhibits Runx2 signalling to impair osteogenic differentiation [9]. It therefore appears that TET molecules may act through direct and indirect mechanisms to regulate BMSC cell fate determination.

Our studies revealed the occupancy of TET2 on bone-associated genes, *RUNX2* and *BMP2,* with *TET2* levels increasing during osteogenic induction across exons and introns, whereas the appearance of 5hmC remained relatively the same, even though TET2 was shown to be a transcriptional activator of these genes based on knockdown experiments. *TET2* knockdown studies showed that the presence of 5hmC on osteogenic and adipogenic promoters was mainly attributed to TET2. A recent study reported that stable expression of the osteoblast transcription factor, *Osterix*, during BMP2-induced osteoblast differentiation involved TET-dependent DNA demethylation and SWI/SNF-associated nucleosome remodelling at the *Osterix* promoter, in mouse embryonic fibroblasts [49]. Therefore, while TET2-mediated conversion of 5mC into 5hmC is needed for transcriptional activation, this occurs in conjunction with other recruited epigenetic enzymes and chromatin remodelling enzymes [49]. It is therefore likely that TET2 can interact with other enzymes to modify the nucleosomes and increase transcription. This is evident in smooth muscle cells, where removal of *TET2* results in a decrease in chromatin accessibility and increased H3K27me3 on *Myocd*, *Srf* and *Myh11* gene loci [10], leading to suppression of smooth muscle differentiation. Genome-wide studies of TET1 deletion in ESC correlated with diminished 5hmC levels and a change in the histone methylation status, supporting the notion that TET proteins and 5hmC can regulate other nucleosome modifications [43, 55]. When considering the gene binding profile of TET1, TET2 and 5hmC in BMSC, we believe that TET-mediated 5hmC is a marker for poised transcription and is present on genes that are active or can be transcriptionally activated in BMSC. This is supported by other studies showing that 5hmC is biased towards activated genes involved in differentiation [56]. Genome-wide studies have discovered that genes with 5hmC at their TSS tend to also be CpG rich and contain bivalent domains, and are more likely to be upregulated during differentiation; thus, 5hmC is likely to contribute to the poised state of activation [57].

While the present study failed to observe any effect of TET3 on BMSC differentiation, there may be some possibility of redundancy between TET molecules. We showed that dual knockdown of both *TET1* and *TET2* in human BMSC was required to significantly decrease global levels of 5hmC and increase 5mC under either normal or osteogenic inductive conditions. However, we failed to observe any additive effects of knocking down both *TET1* and *TET2* on BMSC osteogenic differentiation, when compared to *TET2* knockdown alone. Similar studies examining *TET3* knockdown in combination with *TET2* knockdown showed no significant additive effects on BMSC osteogenic differentiation compared to *TET2* alone (data not shown), implying that TET2 has a dominant effect on osteogenesis.

In an attempt to initiate studies examining the function of TET enzymes during bone disease, we examined expression levels of *TET* molecules in ovariectomised osteoporotic mice. The data demonstrated a significant decrease in expression of *TET1* and *TET2* and overall 5hmC levels in the bones of ovariectomised mice. These results strengthen our conclusions that *TET1* and *TET2* have important functions in 5hmC and bone biology Their concomitant reduction in the murine osteoporotic model also suggests that both TET1 and TET2 could have similar functions in murine bone marrow stromal cells. A recent mouse study showed that depletion of both *TET1* and *TET2* resulted in impaired self-renewal and differentiation of BMSC, which was associated with a significant osteopenia phenotype and supports the observation that TET1 and TET2 expression is reduced in OXV mice leading to decreased 5hmC levels in BMSC and impaired bone formation [9]. Given that both TET1 and TET2 are severely reduced in osteoporosis, pharmacological re-induction of *TET1/TET2* may be of therapeutic benefit to individuals suffering from age-related bone loss or osteoporosis [58].

## Conclusion

**Fig. 10:**
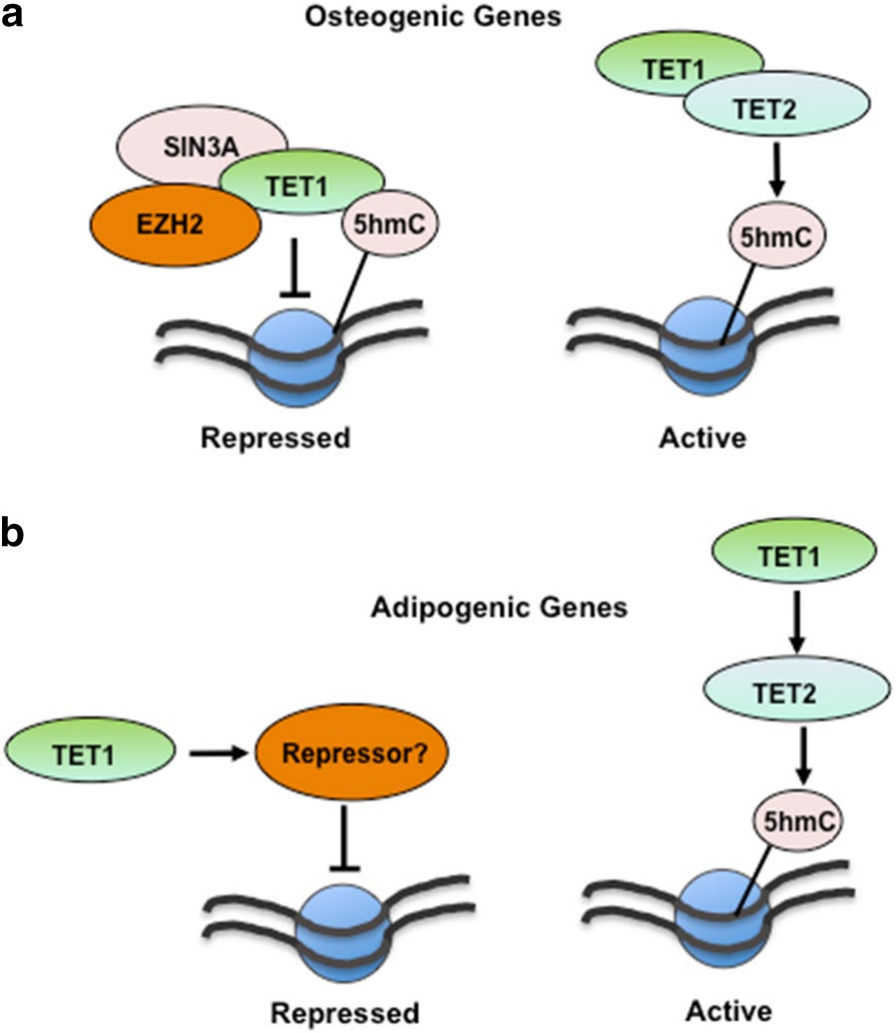
Function of TET1 and TET2 in human MSC lineage determination. **a** 5hmC mark on osteogenic genes is directly attributed to TET2 and to some extent TET1 allowing a permissive transcriptional state. TET1 also represses osteogenic gene transcription presumably by recruiting SIN3A and EZH2, where its removal during osteogenesis activates gene transcription. **b** TET2 is directly responsible for 5hmC on adipogenic genes, allowing for a permissive transcriptional state, where TET1 indirectly represses adipogenic gene transcription

In summary, TET DNA dioxygenases were found to play a critical role in BMSC differentiation. TET1 was found to be an inhibitor of BMSC osteogenic and adipogenic differentiation and appeared to act via an indirect mechanism through the recruitment of other epigenetic modifiers such as SIN3A and EZH2 (Fig. 10). Conversely, TET2 was found to directly promote BMSC osteogenic and adipogenic differentiation. Moreover, both TET1 and TET2 transcript levels were shown to be downregulated during osteoporosis. Targeting TET molecules or their downstream targets may offer new therapeutic strategies to help prevent bone loss and repair following trauma or disease.

## Additional file

**Additional file 1: Figure S1.** TET1 and TET2 contribute to global 5hmC and 5mC demethylation. (a) Human BMSC were treated with siRNA directed to *TET1* (siTET1) or *TET2* (siTET2) alone or in combination (siTET1 + 2), then cultured under normal control media or under osteogenic inductive conditions for 1 week. Genomic DNA was purified and global 5hmC and 5mC levels were measured by ELISA, relative to total input DNA. Data represent mean S.E.M, *n* = 3 BMSC donors, *p < 0.05, one-way ANOVA with multiple comparisons. (b) BMSC were treated with either siTET1 or siTET2 alone or in combination (siTET1 + 2) and then cultured under osteogenic inductive conditions for 3 weeks. Mineral deposits were stained with Alizarin red.

## Abbreviations

BMSC: bone marrow stromal/stem cells
TET: ten-eleven translocation proteins
5mC: 5 methylcytosine
5hmC: 5 hydroxymethylcytosine
OVX: ovariectomised
ESC: embryonic stem cells
HSC: hematopoietic stem cells
H3K4, H3K27: histone 3 lysine 4 or 27
TSS: transcription start site
UTR: untranslated region
5Aza-2: 5 azacytidine

## References

[1] Kouzarides T (2007). Chromatin modifications and their function. Cell.

[2] Tahiliani M, Koh KP, Shen Y, Pastor WA, Bandukwala H, Brudno Y, Agarwal S, Iyer LM, Liu DR, Aravind L (2009). Conversion of 5-methylcytosine to 5-hydroxymethylcytosine in mammalian DNA by MLL partner TET1. Science.

[3] Szwagierczak A, Bultmann S, Schmidt CS, Spada F, Leonhardt H (2010). Sensitive enzymatic quantification of 5-hydroxymethylcytosine in genomic DNA. Nucleic Acids Res.

[4] Hattori N, Imao Y, Nishino K, Hattori N, Ohgane J, Yagi S, Tanaka S, Shiota K (2007). Epigenetic regulation of Nanog gene in embryonic stem and trophoblast stem cells. Genes Cells.

[5] Ito S, D’Alessio AC, Taranova OV, Hong K, Sowers LC, Zhang Y (2010). Role of Tet proteins in 5mC to 5hmC conversion, ES-cell self-renewal and inner cell mass specification. Nature.

[6] Zhao Z, Chen L, Dawlaty MM, Pan F, Weeks O, Zhou Y, Cao Z, Shi H, Wang J, Lin L (2015). Combined loss of Tet1 and Tet2 promotes B cell, but not myeloid malignancies, in mice. Cell Rep.

[7] Delhommeau F, Dupont S, Della Valle V, James C, Trannoy S, Masse A, Kosmider O, Le Couedic JP, Robert F, Alberdi A (2009). Mutation in TET2 in myeloid cancers. N Engl J Med.

[8] Shimoda K, Shide K, Kameda T, Hidaka T, Kubuki Y, Kamiunten A, Sekine M, Akizuki K, Shimoda H, Yamaji T (2015). TET2 mutation in adult T-cell leukemia/lymphoma. J Clin Exp Hematop.

[9] Yang R, Yu T, Kou X, Gao X, Chen C, Liu D, Zhou Y, Shi S (2018). Tet1 and Tet2 maintain mesenchymal stem cell homeostasis via demethylation of the P2rX7 promoter. Nat Commun.

[10] Liu R, Jin Y, Tang WH, Qin L, Zhang X, Tellides G, Hwa J, Yu J, Martin KA (2013). Ten-eleven translocation-2 (TET2) is a master regulator of smooth muscle cell plasticity. Circulation.

[11] Yang R, Qu C, Zhou Y, Konkel JE, Shi S, Liu Y, Chen C, Liu S, Liu D, Chen Y (2015). Hydrogen sulfide promotes Tet1- and Tet2-mediated Foxp3 demethylation to drive regulatory T cell differentiation and maintain immune homeostasis. Immunity.

[12] Dawlaty MM, Breiling A, Le T, Raddatz G, Barrasa MI, Cheng AW, Gao Q, Powell BE, Li Z, Xu M (2013). Combined deficiency of Tet1 and Tet2 causes epigenetic abnormalities but is compatible with postnatal development. Dev Cell.

[13] Dawlaty MM, Breiling A, Le T, Barrasa MI, Raddatz G, Gao Q, Powell BE, Cheng AW, Faull KF, Lyko F (2014). Loss of Tet enzymes compromises proper differentiation of embryonic stem cells. Dev Cell.

[14] Gao Y, Chen J, Li K, Wu T, Huang B, Liu W, Kou X, Zhang Y, Huang H, Jiang Y (2013). Replacement of Oct4 by Tet1 during iPSC induction reveals an important role of DNA methylation and hydroxymethylation in reprogramming. Cell Stem Cell.

[15] Friedenstein A, Thiernfelder S (1980). Stromal mechanocytes of bone marrow: cloning in vitro and retransplantation in vivo. Immunology of bone marrow transplantation.

[16] Pittenger MF, Mackay AM, Beck SC, Jaiswal RK, Douglas R, Mosca JD, Moorman MA, Simonetti DW, Craig S, Marshak DR (1999). Multilineage potential of adult human mesenchymal stem cells. Science.

[17] Owen M, Friedenstein A (1988). Marrow-derived osteogenic precursors. CIba Found Symp.

[18] Gronthos S, Zannettino A, Hay S (2003). Molecular and cellular characterisation of highly purified stromal stem cells derived from human bone marrow. J Cell Sci.

[19] Sacchetti B, Funari A, Michienzi S, Di Cesare S, Piersanti S, Saggio I, Tagliafico E, Ferrari S, Robey PG, Riminucci M (2007). Self-renewing osteoprogenitors in bone marrow sinusoids can organize a hematopoietic microenvironment. Cell.

[20] Nayak A, Muller S (2014). SUMO-specific proteases/isopeptidases: SENPs and beyond. Genome Biol.

[21] Hemming S, Cakouros D, Isenmann S, Cooper L, Menicanin D, Zannettino AC, Gronthos S (2013). EZH2 and KDM6A act as an epigenetic switch to regulate mesenchymal stem cell lineage specification. Stem Cells.

[22] Ye L, Fan Z, Yu B, Chang J, Al Hezaimi K, Zhou X, Park N, Wang C (2012). Histone demethylases KDM4B and KDM6B promotes osteogenic differentiation of human MSCs. Cell Stem Cell.

[23] Zhang F, Xu L, Xu L, Xu Q, Karsenty G, Chen CD (2015). Histone demethylase JMJD3 is required for osteoblast differentiation in mice. Sci Rep.

[24] Zhang F, Xu L, Xu L, Xu Q, Li D, Yang Y, Karsenty G, Chen CD (2015). JMJD3 promotes chondrocyte proliferation and hypertrophy during endochondral bone formation in mice. J Mol Cell Biol.

[25] Komori T, Yagi H, Nomura S, Yamaguchi A, Sasaki K, Deguchi K, Shimizu Y, Bronson R, Gao Y, Inada M (1997). Targeted disruption of Cbfa1 results in a complete lack of bone formation owing to maturational arrest of osteoblasts. Cell.

[26] Ducy P, Zhang R, Geoffroy V, Ridall AL, Karsenty G (1997). Osf2/Cbfa1: a transcriptional activator of osteoblast differentiation. Cell.

[27] Tontonoz P, Hu E, Spiegelman BM (1994). Stimulation of adipogenesis in fibroblasts by PPAR gamma 2, a lipid-activated transcription factor. Cell.

[28] Cartron PF, Nadaradjane A, Lepape F, Lalier L, Gardie B, Vallette FM (2013). Identification of TET1 partners that control its DNA-demethylating function. Genes Cancer.

[29] Isenmann S, Arthur A, Zannettino A, Turner J, Shi S, Glackin C, Gronthos S (2009). Twist family of basic-helix–loop-helix transcription factors mediate human mesenchymal stem cell growth and commitment. Stem Cells.

[30] Fitter S, Vandyke K, Gronthos S, Zannettino AC (2012). Suppression of PDGF-induced PI3 kinase activity by imatinib promotes adipogenesis and adiponectin secretion. J Mol Endocrinol.

[31] Hemming S, Cakouros D, Isenmann S, Cooper L, Menicanin D, Zannettino A, Gronthos S (2014). EZH2 and KDM6A act as an epigenetic switch to regulate mesenchymal stem cell lineage specification. Stem Cells.

[32] Cakouros D, Isenmann S, Hemming SE, Menicanin D, Camp E, Zannettino AC, Gronthos S (2015). Novel basic helix loop helix transcription factor Hes4 antagonizes the function of twist-1 to regulate lineage commitment of bone marrow stromal/stem cells. Stem Cells Dev.

[33] Cakouros D, Isenmann S, Cooper L, Zannettino A, Anderson P, Glackin C, Gronthos S (2012). Twist-1 induces Ezh2 recruitment regulating histone methylation along the Ink4A/Arf locus in mesenchymal stem cells. Mol Cell Biol.

[34] Yamaza T, Miura Y, Bi Y, Liu Y, Akiyama K, Sonoyama W, Patel V, Gutkind Young M, Gronthos S (2008). Pharmacologic stem cell based intervention as a new approach to osteoporosis treatment in rodents. Plos One.

[35] Hemming S, Cakouros D, Codrington J, Vandyke K, Arthur A, Zannettino A, Gronthos S (2017). EZH2 deletion in early mesenchyme compromises postnatal bone microarchitecture and structural integrity and accelerates remodeling. FASEB J.

[36] Nguyen TM, Arthur A, Panagopoulos R, Paton S, Hayball JD, Zannettino AC, Purton LE, Matsuo K, Gronthos S (2015). EphB4 expressing stromal cells exhibit an enhanced capacity for hematopoietic stem cell maintenance. Stem Cells.

[37] D’Ippolito G, Schiller PC, Ricordi C, Roos BA, Howard GA (1999). Age-related osteogenic potential of mesenchymal stromal stem cells from human vertebral bone marrow. J Bone Miner Res.

[38] Neri F, Incarnato D, Krepelova A, Rapelli S, Pagnani A, Zecchina R, Parlato C, Oliviero S (2013). Genome-wide analysis identifies a functional association of Tet1 and Polycomb repressive complex 2 in mouse embryonic stem cells. Genome Biol.

[39] Deplus R, Delatte B, Schwinn MK, Defrance M, Mendez J, Murphy N, Dawson MA, Volkmar M, Putmans P, Calonne E (2013). TET2 and TET3 regulate GlcNAcylation and H3K4 methylation through OGT and SET1/COMPASS. EMBO J.

[40] Williams K, Christensen J, Pedersen MT, Johansen JV, Cloos PA, Rappsilber J, Helin K (2011). TET1 and hydroxymethylcytosine in transcription and DNA methylation fidelity. Nature.

[41] Xu Y, Wu F, Tan L, Kong L, Xiong L, Deng J, Barbera AJ, Zheng L, Zhang H, Huang S (2011). Genome-wide regulation of 5hmC, 5mC, and gene expression by Tet1 hydroxylase in mouse embryonic stem cells. Mol Cell.

[42] Mohn F, Weber M, Rebhan M, Roloff TC, Richter J, Stadler MB, Bibel M, Schubeler D (2008). Lineage-specific polycomb targets and de novo DNA methylation define restriction and potential of neuronal progenitors. Mol Cell.

[43] Wu H, D’Alessio AC, Ito S, Wang Z, Cui K, Zhao K, Sun YE, Zhang Y (2011). Genome-wide analysis of 5-hydroxymethylcytosine distribution reveals its dual function in transcriptional regulation in mouse embryonic stem cells. Genes Dev.

[44] Koh KP, Yabuuchi A, Rao S, Huang Y, Cunniff K, Nardone J, Laiho A, Tahiliani M, Sommer CA, Mostoslavsky G (2011). Tet1 and Tet2 regulate 5-hydroxymethylcytosine production and cell lineage specification in mouse embryonic stem cells. Cell Stem Cell.

[45] Gao J, Ma Y, Fu HL, Luo Q, Wang Z, Xiao YH, Yang H, Cui DX, Jin WL (2016). Non-catalytic roles for TET1 protein negatively regulating neuronal differentiation through srGAP3 in neuroblastoma cells. Protein Cell.

[46] Dawlaty MM, Ganz K, Powell BE, Hu YC, Markoulaki S, Cheng AW, Gao Q, Kim J, Choi SW, Page DC (2011). Tet1 is dispensable for maintaining pluripotency and its loss is compatible with embryonic and postnatal development. Cell Stem Cell.

[47] Li X, Yue X, Pastor WA, Lin L, Georges R, Chavez L, Evans SM, Rao A (2016). Tet proteins influence the balance between neuroectodermal and mesodermal fate choice by inhibiting Wnt signaling. Proc Natl Acad Sci USA.

[48] Yoo Y, Park JH, Weigel C, Liesenfeld DB, Weichenhan D, Plass C, Seo DG, Lindroth AM, Park YJ (2017). TET-mediated hydroxymethylcytosine at the Ppargamma locus is required for initiation of adipogenic differentiation. Int J Obes.

[49] Sepulveda H, Villagra A, Montecino M (2017). Tet-mediated DNA demethylation is required for SWI/SNF-dependent chromatin remodeling and histone-modifying activities that trigger expression of the Sp7 osteoblast master gene during mesenchymal lineage commitment. Mol Cell Biol.

[50] Li X, Yao B, Chen L, Kang Y, Li Y, Cheng Y, Li L, Lin L, Wang Z, Wang M (2017). Ten-eleven translocation 2 interacts with forkhead box O3 and regulates adult neurogenesis. Nat Commun.

[51] Zhong J, Li X, Cai W, Wang Y, Dong S, Yang J, Zhang J, Wu N, Li Y, Mao F (2017). TET1 modulates H4K16 acetylation by controlling auto-acetylation of hMOF to affect gene regulation and DNA repair function. Nucleic Acids Res.

[52] Hon GC, Song CX, Du T, Jin F, Selvaraj S, Lee AY, Yen CA, Ye Z, Mao SQ, Wang BA (2014). 5mC oxidation by Tet2 modulates enhancer activity and timing of transcriptome reprogramming during differentiation. Mol Cell.

[53] Taylor SE, Li YH, Smeriglio P, Rath M, Wong WH, Bhutani N (2016). Stable 5-hydroxymethylcytosine (5hmC) acquisition marks gene activation during chondrogenic differentiation. J Bone Miner Res Off J Am Soc Bone Miner Res.

[54] Rao LJ, Yi BC, Li QM, Xu Q (2016). TET1 knockdown inhibits the odontogenic differentiation potential of human dental pulp cells. Int J Oral Sci.

[55] Wu H, D’Alessio AC, Ito S, Xia K, Wang Z, Cui K, Zhao K, Sun YE, Zhang Y (2011). Dual functions of Tet1 in transcriptional regulation in mouse embryonic stem cells. Nature.

[56] Serandour AA, Avner S, Oger F, Bizot M, Percevault F, Lucchetti-Miganeh C, Palierne G, Gheeraert C, Barloy-Hubler F, Peron CL (2012). Dynamic hydroxymethylation of deoxyribonucleic acid marks differentiation-associated enhancers. Nucleic Acids Res.

[57] Pastor WA, Pape UJ, Huang Y, Henderson HR, Lister R, Ko M, McLoughlin EM, Brudno Y, Mahapatra S, Kapranov P (2011). Genome-wide mapping of 5-hydroxymethylcytosine in embryonic stem cells. Nature.

[58] Liu Y, Yang R, Liu X, Zhou Y, Qu C, Kikuiri T, Wang S, Zandi E, Du J, Ambudkar IS (2014). Hydrogen sulfide maintains mesenchymal stem cell function and bone homeostasis via regulation of Ca(2 +) channel sulfhydration. Cell Stem Cell.

