# Supplementary figures and images for: Specific functions of TET1 and TET2 in regulating mesenchymal cell lineage determination
Source: Epigenetics Chromatin. 2019 Jan 3;12:3. doi: 10.1186/s13072-018-0247-4 (PMC6317244; doi:10.1186/s13072-018-0247-4)

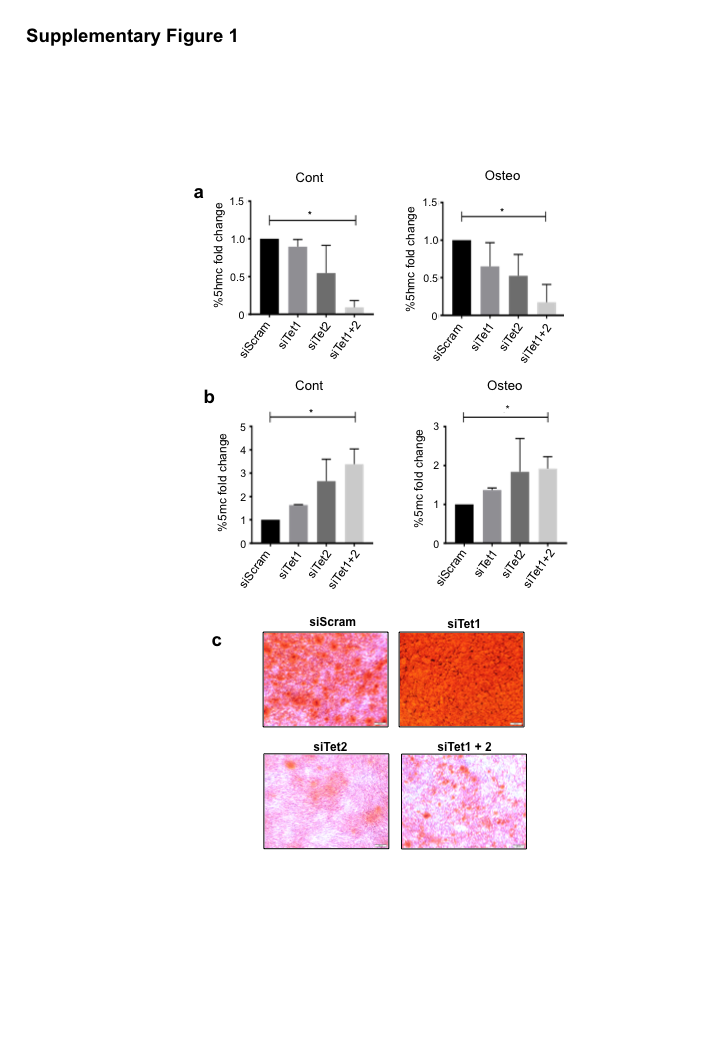

Supplement: Supplementary file 1 — Additional file 1: Figure S1. TET1 and TET2 contribute to global 5hmC and 5mC demethylation. (a) Human BMSC were treated with siRNA directed to TET1 (siTET1) or TET2 (siTET2) alone or in combination (siTET1 + 2), then cultured under normal control media or under osteogenic inductive conditions for 1 week. Genomic DNA was purified and global 5hmC and 5mC levels were measured by ELISA, relative to total input DNA. Data represent mean S.E.M, n = 3 BMSC donors, *p < 0.05, one-way ANOVA with multiple comparisons. (b) BMSC were treated with either siTET1 or siTET2 alone or in combination (siTET1 + 2) and then cultured under osteogenic inductive conditions for 3 weeks. Mineral deposits were stained with Alizarin red. [file 13072_2018_247_MOESM1_ESM.tiff]
